# Analysis of 427 genomes reveals moso bamboo population structure and genetic basis of property traits

**DOI:** 10.1038/s41467-021-25795-x

**Published:** 2021-09-15

**Authors:** Hansheng Zhao, Shuai Sun, Yulong Ding, Yue Wang, Xianghua Yue, Xiao Du, Qiang Wei, Guangyi Fan, Huayu Sun, Yongfeng Lou, Huanming Yang, Jian Wang, Xun Xu, Lichao Li, Kebin Yang, Hao Xu, Jiongliang Wang, Chenglei Zhu, Sining Wang, Xuemeng Shan, Yinguang Hou, Yu Wang, Benhua Fei, Xin Liu, Zehui Jiang, Zhimin Gao

**Affiliations:** 1 Institute of Gene Science and Industrialization for Bamboo and Rattan Resources, International Center for Bamboo and Rattan, 100102 Beijing, China; 2 Key Laboratory of National Forestry and Grassland Administration/Beijing for Bamboo & Rattan Science and Technology, 100102 Beijing, China; 3 BGI-Qingdao, BGI-Shenzhen, 266555 Qingdao, China; 4 China National GeneBank, BGI-Shenzhen, 518120 Shenzhen, China; 5 College of Life Sciences, University of Chinese Academy of Sciences, 100049 Beijing, China; 6 Bamboo Research Institute, Nanjing Forestry University, 210037 Nanjing, China; 7 BGI-Shenzhen, 518083 Shenzhen, China; 8 State Key Laboratory of Agricultural Genomics, BGI-Shenzhen, 518083 Shenzhen, China; 9 Guangdong Provincial Academician Workstation of BGI Synthetic Genomics, BGI-Shenzhen, 518120 Shenzhen, China; 10 James D. Watson Institute of Genome Science, 310008 Hangzhou, China; 11 BGI-Beijing, BGI-Shenzhen, 100101 Beijing, China; 12 BGI-Fuyang, BGI-Shenzhen, 236009 Fuyang, China

**Keywords:** Genome-wide association studies, DNA sequencing, Natural variation in plants, Plant evolution

## Abstract

Moso bamboo (*Phyllostachys edulis*) is an economically and ecologically important nontimber forestry species. Further development of this species as a sustainable bamboo resource has been hindered by a lack of population genome information. Here, we report a moso bamboo genomic variation atlas of 5.45 million single-nucleotide polymorphisms (SNPs) from whole-genome resequencing of 427 individuals covering 15 representative geographic areas. We uncover low genetic diversity, high genotype heterozygosity, and genes under balancing selection underlying moso bamboo population adaptation. We infer its demographic history with one bottleneck and its recently small population without a rebound. We define five phylogenetic groups and infer that one group probably originated by a single-origin event from East China. Finally, we conduct genome-wide association analysis of nine important property-related traits to identify candidate genes, many of which are involved in cell wall, carbohydrate metabolism, and environmental adaptation. These results provide a foundation and resources for understanding moso bamboo evolution and the genetic mechanisms of agriculturally important traits.

## Abstract (web summary)

Moso bamboo is an economically and ecologically important nontimber forestry species. Here, the authors analyze 427 genomes collected from 15 representative geographic areas, and identify genes under balancing selection, putative patterns of historic demography, and candidate genes associated with important traits.

## Introduction

Moso bamboo (*Phyllostachys edulis*) is the most important bamboo species worldwide, accounting for ~74% of the total bamboo-growing area (4.68 million ha)^1^, with production corresponding to 5 billion US dollars annually in China^2^. It is primarily an asexually reproducing perennial grass in subtropical areas and is mainly distributed in southern China (a relatively independent geographic population has been established in the area between ~23°30′ to 32°20′ N and 104°30′ to 122° E)^3^, and its growth status varies with latitude, longitude, and topography^4^. Human activities and environmental factors have caused some disturbance to spontaneous vegetation, resulting in habitat deterioration and germplasm loss^5^. A genome-scale investigation of the genetic diversity, population differentiation, and spatial structure of moso bamboo across the entire distribution range in China are essential for designing and implementing appropriate conservation strategies to harness its natural and domesticated biodiversity.

Recent studies on the genetic diversity of moso bamboo have been performed using various types of molecular markers, such as random amplified polymorphic DNA (RAPD)^6^, amplified fragment-length polymorphism (AFLP)^7^, inter simple sequence repeat (ISSR)^8^, and simple sequence repeat (SSR) markers^9^. However, a thorough understanding of the genetic diversity and population structure of moso bamboo is lacking due to the unavailability of a more comprehensive population analysis of genome-wide variations, especially when the reference genome is available^10,11^. Whole-genome resequencing (WGRS) has been widely applied to important plants to understand the extent/patterns of genetic variation and linkage disequilibrium and to reveal the unidentified genetic potential for critical agronomic traits^12–15^. With the advent of next-generation sequencing technology, WGRS has greatly facilitated the identification of sites associated with phenotypic traits^16,17^, such as plant disease resistance, yield, and property traits. Carrying out WGRS in moso bamboo will help to identify and utilize variants of different frequencies in the population that may contribute to crucial phenotypes, including bamboo property traits.

In this study, we sequence 427 moso bamboo individuals from 15 representative geographic distribution areas of moso bamboo in China to identify genome-wide variations, including single-nucleotide polymorphisms (SNPs), small insertions, and deletions (InDels), structural variations (SVs), and copy number variations (CNVs). These variations are further analyzed to understand genome features and population structure to aid in further research and applications. The identified variations provide insight into the origin and evolutionary history of moso bamboo and reveal possible genetic loci related to the agronomic traits of moso bamboo.

## Results

### Large-scale WGRS revealed low genomic diversity in the moso bamboo population

**Fig. 1:**
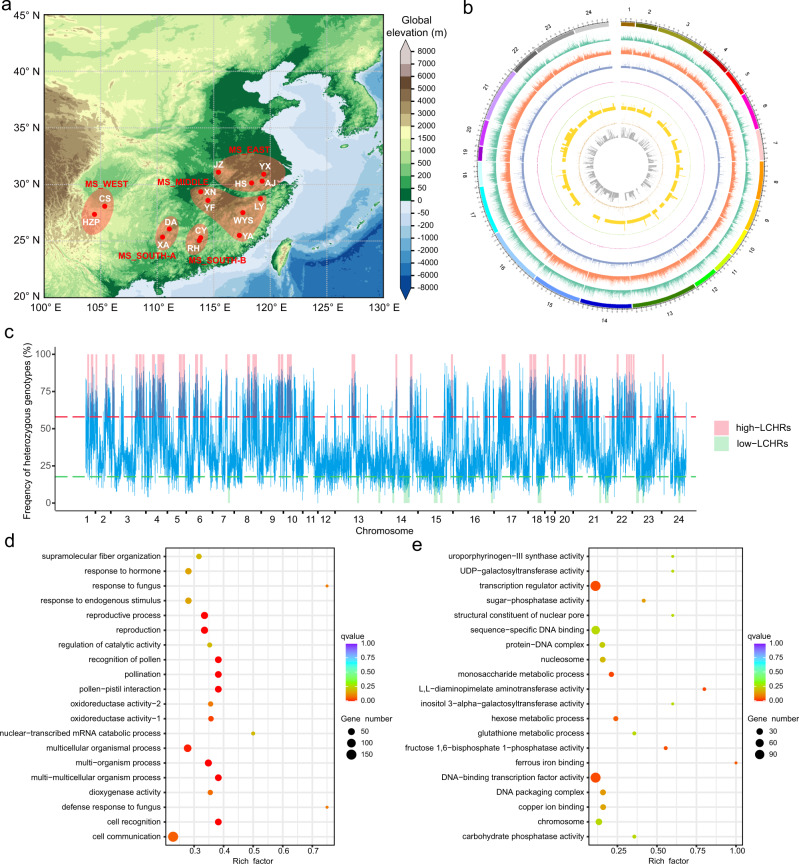
The landscape of sampling and variants in sequenced moso bamboo individuals. **a** The sampling locations of 15 major moso bamboo geographic areas are indicated with red points, and five empirically assigned phylogenetic groups according to the genetic structure and large-scale geographic distribution are represented in light shades. The map was drawn based on ETOPO2v2c Global Gridded 2-min elevation and bathymetric data (doi: 10.7289/V5J1012Q). **b** Circos plot for the visualization of different types of detected variants in the moso bamboo population at the genome-wide level. The tracks from outside to the inside represent the density of SNPs (single-nucleotide polymorphisms), InDels (small insertions and deletions), large deletions (DELs), insertions (INSs), inversions (INVs), intrachromosomal translocations (ITX), gained copy number variations (CNVs), and lost CNVs. Variant density was calculated in non-overlapping 100-kb window intervals. **c** Heterozygous genotype frequency in non-overlapping 200-kb windows genome wide. The red and green dashed lines represent the thresholds of high and low heterozygous genotype frequencies, respectively. The long continuous heterozygous SNPs clustered regions of high frequency (high-LCHRs) and long continuous heterozygous SNPs clustered regions of low frequency (low-LCHRs) are shaded in red and green, respectively. **d** The dot plot shows the gene ontology (GO) enrichment of genes located in high-LCHRs. **e** The dot plot shows the GO enrichment of genes located in low-LCHRs. The color of the points represents the Benjamini–Hochberg corrected *p*-value, and the size of points represents the number of genes. Rich factor is the ratio of the number of interested genes annotated in this GO term to all genes in this GO term. Source data underlying **b**–**e** are provided as a Source Data file.

A total of 427 representative moso bamboo individuals from 15 major geographic distribution areas were selected for WGRS according to an SSR-based phylogenetic tree^9^ (Fig. 1a, Supplementary Table 1 and “Methods” section). A sample from a closely related species (*Phyllostachys kwangsiensis*) was also sequenced to be used as the outgroup. In total, we generated 16.60 Tb data (55.34 billion read pairs) and mapped these data to the reference genome^10^, resulting in an average sequencing depth of 20.91× (Supplementary Data 1). Based on the mapping results, we ultimately identified 5.45 million high-quality SNPs (Fig. 1b and Supplementary Table 2, Supplementary Fig. 1) and 1.08 million small InDels (<50 bp, Supplementary Table 3). Thus, the global SNP density we calculated was one SNP per 351 bp on average (Supplementary Table 2), which was much lower than that observed for *Arabidopsis* (one SNP per 11 bp)^18^ and rice (one SNP per 16 bp)^19^ (we sequenced fewer individuals than those in *Arabidopsis* and rice). We found that approximately 93.97% of these SNPs are located in intergenic regions, compared to 3.18% in intronic regions and 2.85% in coding regions (Supplementary Table 4). The nonsynonymous-to-synonymous substitution ratio among all biallelic SNPs was 1.50 (Supplementary Table 4), which was higher than that of *Arabidopsis*^20^ (0.83), rice^21^ (1.20), and soybean^21^ (1.36). Overall, we found relatively low whole-genome diversity in the moso bamboo population, indicating a possible low effective population size and small genetic pool to be used for future breeding purposes.

In order to comprehensively depict the genomic variations in moso bamboo, we also detected the structural variants (SVs) and copy number variations (CNVs) (Supplementary Fig. 1). We identified SVs including insertions (INSs), deletions (DELs), inversions (INVs), and intrachromosomal translocations (ITXs). A total of 21,042 SVs were identified in these 427 individuals and the average number of SVs of each individual was 4120, ranging from 2508 to 8380 (Supplementary Fig. 2 and Supplementary Table 5, Supplementary Data 2). To infer the possible functional importance of the SVs, we identified 7483 genes to be overlapped with these SVs thus they might be affected by SVs. Among these genes, 1146 were overlapped with two or more types of SVs (Supplementary Data 3), indicating very high variabilities in these genes caused by SVs. We further carried out gene function enrichment analysis (Supplementary Data 4) for these highly variable genes to find significant enrichment in plant–pathogen interaction and secondary metabolism (e.g., sesquiterpenoid and triterpenoid biosynthesis, Benjamini–Hochberg corrected *p*-value < 0.01). This reflected the functional importance of the SVs, indicating their involvement in pathogen responses. Similarly, we detected a total of 168,700 CNVs, and 92,684 CNVs per moso bamboo individual on average, with a minimum of 81,156 and a maximum of 104,588 CNVs (Supplementary Fig. 2 and Supplementary Data 2). We found the 3306 genes overlapped with CNVs (Supplementary Data 3) were also most significantly enriched in the pathway of plant–pathogen interaction (Supplementary Data 5), which further implied the functional importance of the SVs and CNVs. These different types of genome-wide sequence variations can serve as an important resource, and future studies on these SVs and the related genes would benefit to understand molecular mechanisms of moso bamboo adaptation and breeding.

### Distribution of heterozygous genotypes and their possible functional significance

Despite the low diversity compared with other species, we found a notably high genotype heterozygosity ratio of 18.33 at the individual level (i.e., among the identified variation loci in each individual, 94.82% were heterozygous on average, as shown in Supplementary Data 6), which was 13 and 52 times higher than the ratios in Mei^22^ (*Prunus mume*, 1.44) and rice^19^ (*Oryza sativa*, 0.35), respectively. During asexual reproduction, rare somatic mutations can accumulate, which would result in the accumulation of low-frequency heterozygous genotypes (no more than 10% of individuals with heterozygous genotypes in sequenced individuals were indicated hereafter as low-frequency heterozygous genotypes). In the moso bamboo population, low-frequency heterozygous genotypes were identified on 46.42% of the detected SNP sites, which was as expected (Supplementary Fig. 3). We thus propose that low-frequency somatic mutations have extensively occurred in moso bamboo, a substantial proportion of which have been inherited and spread through asexual reproduction without recombination and segregation, resulting in large quantities of heterozygous genotypes of SNPs. However, we found that 34.55% of SNP sites were also at the status of high-frequency heterozygous genotypes (no less than 90% of individuals with heterozygous genotypes in sequenced individuals) (Supplementary Fig. 3), which indicated that a majority of the individuals were heterozygous at those loci. For both the low-frequency and high-frequency heterozygous genotypes, we depicted their distribution along the genome, revealing that they were almost randomly distributed (Supplementary Fig. 4).

Since somatic mutations should occur randomly and the heterozygous genotypes are distributed randomly, we further identified the long continuous areas with uniform patterns of heterozygous genotypes, which may reflect subtle gene functions underlying evolution or adaptation. In total, we identified 38 long continuous heterozygous SNPs clustered regions of high frequency (high-LCHRs; Fig. 1c and Supplementary Data 7) with a total length of 287.6 Mb (see “Methods” section) and containing 8634 genes. We analyzed the gene ontology (GO) annotation of these genes and found them to be significantly (Benjamini–Hochberg corrected *p*-value < 0.05, method described in “Methods” section and a previous publication^23^) enriched in biological processes of pollen and reproduction recognition, as well as defense responses to fungi and bacteria (Fig. 1d and Supplementary Data 8), reflecting the functional importance of the high-frequency SNPs. We further identified 15 long continuous heterozygous SNPs clustered regions of low frequency (low-LCHRs; Fig. 1c and Supplementary Data 9) with a total length of 115.6 Mb. We also found that 4162 genes in these low heterozygosity regions were significantly enriched in amino acid biosynthesis processes and transcription regulator activities (Benjamini–Hochberg corrected *p*-value < 0.05) (Fig. 1e and Supplementary Data 10), indicating potentially essential functions of these genes. Thus, overall, we found that in moso bamboo, somatic mutations resulted in heterozygosity and might be maintained in some regions while eliminated in others, likely playing an important role in population evolution and adaptation.

### Balancing selection as an evolutionary force probably contributes to environmental adaptation

**Fig. 2:**
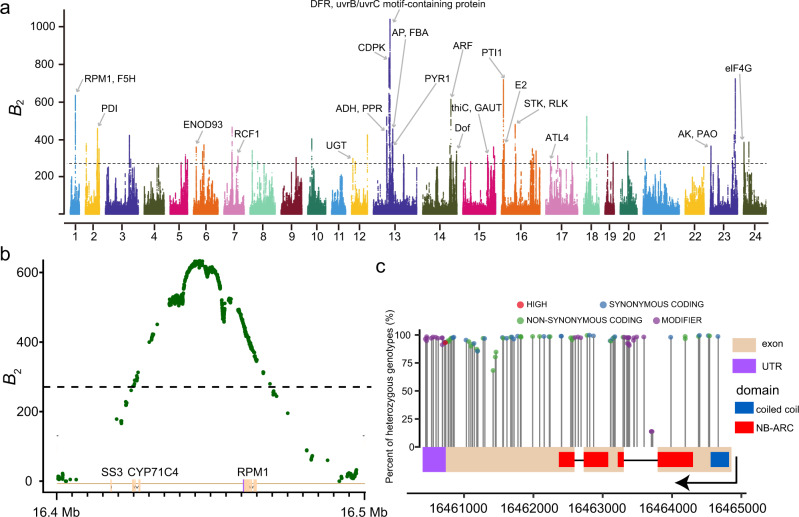
Balancing selection in the moso bamboo population underlies adaptation. **a** Regions of balancing selection detected in the whole moso bamboo population based on *B*_2_ statistics. The dashed line represents the significance level of the top 0.5%. The candidate genes under balancing selection are indicated using arrows with gene names (the full names of these genes are listed in Supplementary Data 13). **b** Enlarged diagram of *B*_2_ statistics around the disease resistance gene *RPM1*. The dashed line represents the genome-wide significance level of the top 0.5%. **c** The diagram shows the SNPs and heterozygous genotype frequency in the *RPM1* gene region (UTR untranslated region, NB-ARC nucleotide-binding adaptor shared by APAF-1, certain R gene products, and CED-4). The function of SNPs was defined and predicted by SnpEff. Source data underlying **b** and **c** are provided as a Source Data file.

Despite the observed low divergence, we sought to uncover the genetic mechanisms underlying their successful adaptation to the environment within a short period and with limited genetic variations. Firstly, we detected potential positive selection signals in the whole population based on a combination of the composite likelihood rate (CLR)^24^ and derived allele frequency (DAF)^25^. We only found two potential regions under positive selection at the highest 1% significance level with one gene of unknown function (Supplementary Fig. 5 and Supplementary Table 6), implying that positive selection might not exert a major effect on moso bamboo population adaptation. Then, we looked for the genomic signatures of balancing selection using *B*_2_ statistics^26^, and found 83 significant peaks containing 120 genes at the highest 0.5% significance level (Fig. 2a and Supplementary Data 11), revealing a higher contribution of balancing selection. Furthermore, GO enrichment of these 120 genes under balancing selection was related to oxidation-reduction processes (Benjamini–Hochberg corrected *p*-value < 0.05), which play an essential role in responding to the environment^27,28^ (Supplementary Data 12). Specifically, 30 genes were involved in disease resistance or environmental adaptation, including genes encoding the disease resistance protein RPM1, dihydroflavonol-4-reductase (DFR), and a pentatricopeptide repeat (PPR) protein (Fig. 2a and Supplementary Data 13). Examining genetic variations within the disease resistance gene *RPM1* (Fig. 2b), we found all detected SNPs to be high-frequency heterozygous SNPs (Fig. 2c), revealing a distinct, significant signature of balancing selection. Balancing selection on immune-related genes might maintain extraordinary genetic diversity within a population and serve as an evolutionary basis for the continuous antagonistic coevolution between hosts and parasites, which has been confirmed in other plants^28–30^. We also found a majority of genes under balancing selection overlapped with the identified high-LCHRs (Supplementary Fig. 6), indicating that the landscape of high-LCHRs might be partly shaped by balancing selection. Thus, balancing selection is proposed to play an important role in the environmental adaptation of moso bamboo populations.

### Discovering five phylogenetic groups and their evolution

**Fig. 3:**
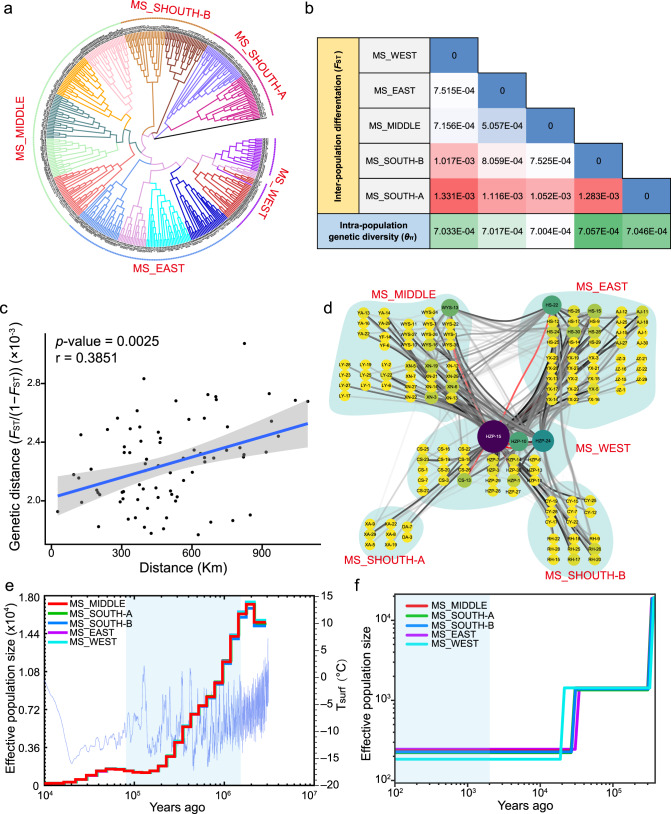
Overall population structure landscape and the inferred population demographic history. **a** Rooted neighbor-joining phylogenetic tree of 427 moso bamboo individuals. The differently colored lines represent the fifteen geographic areas, and the differently colored dotted lines nearby represent five groups that were empirically assigned in our study. **b** The genetic diversity (*θ*π) and population differentiation (*F*_ST_) matrix of the five groups. The colors and numbers in the cells of the matrix represent the *F*_ST_ values. The colors and numbers in the cells below the *F*_ST_ matrix represent the genetic diversity (*θ*π). **c** Results of the Mantel test of the relationship between geographic distance and genetic distance with MS_WEST excluded, and *p*-value was calculated using a one-sided Mantel test with 9999 permutations. The blue line is fitted by the linear regression between genetic distance and geographic distance on the basis of ordinary least squares in the function “geom_smooth” from ggplot2. The gray error band represents the 95% CI (confidence interval). **d** The connection of individuals with the lowest 1% pairwise genetic distances. The size and color of circles represent the degree of connectivity to a node. The lines in different colors indicated values of Hamming distance (genetic distance), with red indicating the shortest distance and for the others, darker colors indicate shorter distances and lighter colors indicate longer distances. **e** The demographic history of the five groups was inferred separately using the pairwise sequential Markovian coalescent (PSMC) method. The blue line represents the historical surface temperatures (*T*_surf_), and the light blue shade indicates the bottleneck experienced during the last glacial period (LGP, 115,000–11,700 years ago). **f** The demographic history was inferred using SMC++. The LGP was shaded in light blue, and the reduction without a rebound in the effective population size during the last 2000 years is shaded in light green. The results were scaled to real-time by assuming a generation time of 67 years and a mutation rate of 8.51 × 10^−8^ per generation. Source data underlying **c** and **d** are provided as a Source Data file.

To reveal the natural population structure of moso bamboo, we applied a subset of 1,432,873 SNPs (linkage disequilibrium pruned) to elucidate the genetic population structure (Supplementary Table 2). The admixture with multiple random seeds showed the best model of *K* = 1, and the unstable ancestry population allocation, indicating the rare slight population differentiation so that we could not reliably define some distinctly genetic subpopulations (Supplementary Figs. 7–11). However, the obtained neighbor-joining (NJ) tree and principal component analysis (PCA) were largely consistent with the geographic distribution, in which most individuals from the same location were clustered into the same clade (Fig. 3a and Supplementary Fig. 12). According to the established phylogenetic tree and the geographic separation distances, we then empirically integrated the 427 individuals from the 15 geographic areas into five phylogenetic groups (Fig. 1a and Fig. 3a), including the east group (MS_EAST), the center group (MS_MIDDLE), the west group (MS_WEST), the south group A (MS_SOUTH-A), and the south group B (MS_SOUTH-B). We found that the genetic diversities (*θ*π) of these phylogenetic groups were low, ranging from 7.00 × 10^−4^ to 7.06 × 10^−4^, and the pairwise Wright’s *F* statistics (*F*_ST_) among groups ranged from 7.16 × 10^−4^ to 1.33 × 10^−3^, reflecting relatively low population differentiation (Fig. 3b and Supplementary Table 7), consistent with our population structure results. Additionally, we found that the MS_SOUTH-A group from Hunan Province with the relatively highest *F*_ST_ to other groups, and was closest to the outgroup (Fig. 3a, b and Supplementary Table 7), suggesting that Hunan Province may represent the recent origin of moso bamboo.

To further depict the relationships among these phylogenetic groups, we performed isolation-by-distance (IBD) analysis and found a significantly positive correlation between geographic distance and genetic distance for the whole moso bamboo population using the Mantel test (*p*-value = 0.0025, *r* = 0.3851, after excluding individuals from HZP and CS) (Fig. 3c), indicating that the limitation of geographic dispersal might be an important factor in the formation of the current genetic population structure. Since MS_WEST was located in a separate geographic region distant from the others (Fig. 1a), we observed no significant correlation (*p*-value = 0.5723, *r* = −0.0512) with MS_WEST included (Supplementary Fig. 13). Meanwhile, MS_WEST was genetically placed on the innermost phylogenetic clade (Fig. 3a) with a relatively low intrapopulation relatedness (Supplementary Fig. 14), implying that MS_WEST probably originated from the most recent colonization. We further constructed an individual-level relatedness network based on the identity-by-state (IBS) genetic distance and found that one MS_WEST individual (HZP-15) constituted a hub that closely connected MS_EAST individuals with the other MS_WEST individuals (Fig. 3d). Thus, we concluded that MS_WEST was the most recently evolved group possibly evolved in a single-origin event from MS_EAST, further illustrating the relationship and history of moso bamboo groups.

### The inferred ancient population bottleneck and recently small population without a rebound are possibly related to climate change and human activities

We noticed low genetic diversity and extremely positive Tajima’s *D* in the moso bamboo population (Supplementary Table 7), so we carried out a demographic analysis to infer historical population changes that resulted in the current population. First, we used the pairwise sequential Markovian coalescent (PSMC)^31^ to investigate the trends of changes in the relatively remote history. As expected, we obtained unsegregated PSMC curves for individuals from five phylogenetic groups (Fig. 3e), all of which showed a rapid decline in the effective population size (Ne) of the moso bamboo population during the last glacial period (115,000–11,700 years ago). The low temperatures and abrupt climate changes during that time^32^ may have led to this substantial bottleneck of the moso bamboo population (Fig. 3e). We then applied the sequential Markov coalescent plenty of unlabeled samples (SMC++)^33^ to reveal a more recent population history, and the results indicated that the population size was extremely small recently without a rebound in recent two thousand years (Fig. 3f), possibly due to human activities, which affected its habitat^34^. The low population-level genetic diversity and the intense population size bottlenecks closely related to climate change and human activities identified in this study provide hints for the further design and implementation of appropriate conservation strategies and the utilization of biodiversity in natural and domesticated bamboos.

### Genome-wide association studies of property-related traits

**Fig. 4:**
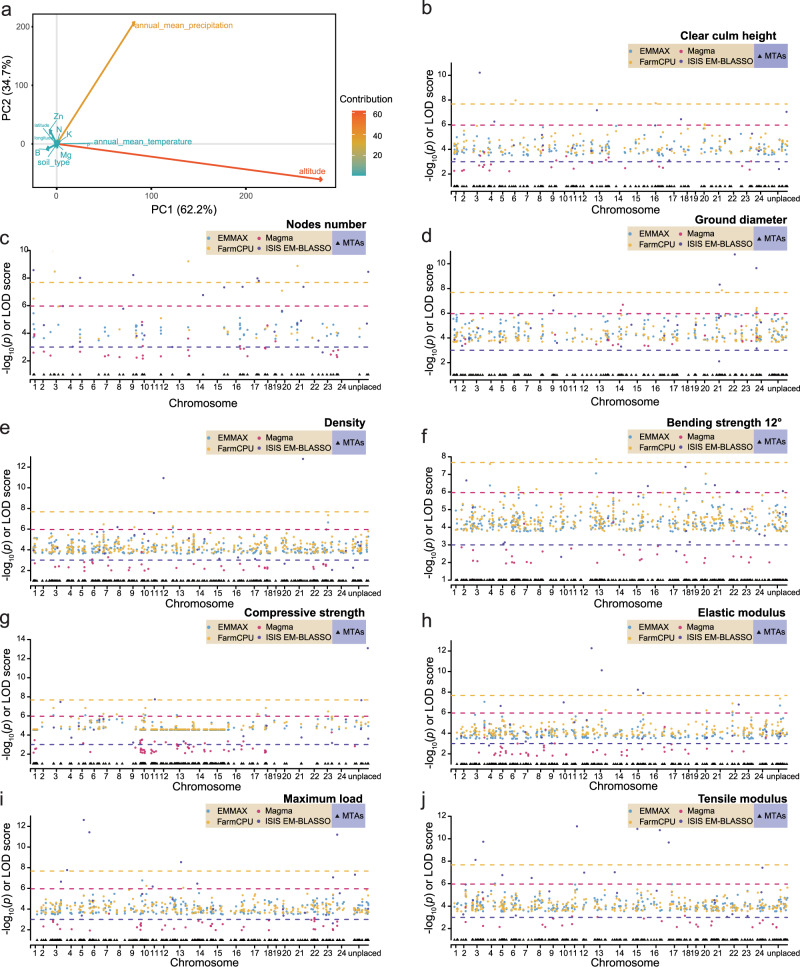
GWAS of important property traits. **a** Principal component analysis (PCA) of 12 environmental factors in which the depth of color represents the contribution of the variable. **b**–**j** Manhattan plot of marker-trait associations (MTAs) identified using statistical approaches for nine traits, including a clear culm height, node number, ground diameter, density, bending strength (12°), compressive strength, elastic modulus, maximum load, and tensile modulus. The statistical approaches include EMMAX, efficient mixed-model association expedited; FarmCPU, fixed and random model circulating probability unification with high statistical power; Magma, gene-level association; ISI EM-BLASSO: based multi-locus association. The *p*-value threshold was 2.10 × 10^−8^, 2.10 × 10^−8^, and 1.08 × 10^−6^ for EMMAX, FarmCPU, and Magma by Bonferroni correction method, separately. The log of odds (LOD) threshold for ISIS EM-BLASSO method is 3 as suggested. For simplicity and clarity, only candidate SNPs were plotted. The MTAs are indicated by black triangles in the lower part of the figure. Source data are provided as a Source Data file.

To explore the association between genetic variations and property-related traits in moso bamboo, we conducted a genome-wide association study (GWAS) to identify associated variations and possibly related genes. Based on previous studies^35–38^, we measured nine traits closely related to properties, collectively referred to as property-related traits in this study (Supplementary Data 14). These traits included morphological features (clear culm height, node number, and ground diameter) and physical (density) and mechanical properties (compressive strength, bending strength 12°, elastic modulus, maximum load, and tensile modulus). Data on twelve environmental factors (details in the “Methods” section) were also collected to control the effects of these environmental factors on the studied traits (Supplementary Data 15). PCA showed that the most influential environmental factors were altitude, annual average precipitation, and annual average temperature, for which the top three principal components (PCs) accounted for more than 99% of the overall variation (Fig. 4a and Supplementary Fig. 15).

We retained 190 samples and 2,915,210 SNPs for GWAS after a series of quality control steps (see “Methods” section and Supplementary Table 9). Based on the characteristics of the data after quality control, we implemented a comprehensive strategy of GWAS (see “Methods” section). For nine phenotypes, the number of significant marker-trait associations (MTAs) detected ranged from 63 to 328 (see “Methods” section and Supplementary Fig. 16), and the number of candidate genes ranged from 43 to 203 (Table 1, Fig. 4b–j and Supplementary Data 16–24). We identified 104 candidate genes to be related to the cell wall, carbohydrate metabolism, and environmental adaptation (Supplementary Data 25). For example, two genes of *PH02Gene04629.t1* and *PH02Gene48149.t1*, both annotated as encoding cinnamoyl-CoA reductase (CCR), which catalyze the conversion of cinnamoyl-CoAs to the corresponding cinnamaldehydes and were reported to cause significant changes in lignin levels and composition^39^. Another example is the *PH02Gene46399.t1* gene, encoding a member of the GRAS (gibberellic acid insensitive, repressor of GAI, and scarecrow) family. Its reciprocal best BLAST hit of *AT1G07530.1* showed that this gene plays a role in adaptation to the environment^40^. Finally, the co-expression network showed that some property trait-associated genes were linked, suggesting that the genes controlling complex traits were often interconnected in regulatory networks (Supplementary Fig. 17). These identified signals and the candidate genes could be further used to improve the efficiency of breeding and aid future genetic mechanism research.

**Table 1:** Summary of the GWAS results.

Phenotypes	Number of MTAs	Number of interested genes	Environmental suitability-related genes	Cell wall-related genes	Carbohydrate metabolism-related genes
Bending strength 12°	302	122	10	1	2
Compressive strength	328	203	14	6	5
Density	263	127	8	9	3
Elastic modulus	217	114	6	2	2
Ground diameter	178	77	7	3	3
Maximum load	210	85	3	1	0
Nodes number	63	43	6	1	0
Tensile modulus	255	89	7	3	3
Clear culm height	146	76	3	2	0

*GWAS* genome-wide association study, *MTAs* marker-trait associations.

## Discussion

Moso bamboo is an essential nontimber economic forest product and exhibits strong environmental adaptability, excellent growth potential, and a wide distribution^41^. Here we sequenced 427 representative moso bamboo individuals at the whole-genome level, thereby revealing the comprehensive population genetic diversity of this mainly asexually reproducing species. We identified genomic variations, which can be further used for future studies and applications, along with unique genomic features of high heterozygosity of genotypes resulting from asexual reproduction and probably resulting in specific functions. Despite the heterozygosity introduced randomly through somatic mutations, we found that much of the heterozygosity could be maintained and fixed in the population because of the possible functional significance through the asexual reproduction process. With its low sexual reproduction rate, long flowering cycle, and unpredictable flowering period, there have been limited studies on moso bamboo selection and breeding, and current research is focusing on cultivation and interspecific hybridization^4^. With representative individuals as germplasms, along with comprehensive genetic variations as digital germplasms, future research and breeding efforts might be more effective.

Our analysis highlights low intrapopulation genetic diversity and interpopulation heterogeneity, the historical bottleneck, and a recently reduced effective population size in moso bamboo. Therefore, conservation strategies should focus on protecting its populations, especially those with high genetic variability (e.g., MS_SOUTH-A) to prevent profound damage to the species. Additionally, abundant specific germplasms provide a source of great variation for establishing ecological moso bamboo forests, and unique alleles may result from populations adapted to their local environments. Such alleles have great value for the maintenance and evolution of the species in unfavorable subsistence environments. Moreover, as a mainly asexual species, the collection of moso bamboo genetic resources will not damage the original distribution. Therefore, efforts aimed at the ex-situ conservation and utilization of the significant germplasms of this species in various populations should be intensified.

We detected abundant genetic variation among individuals in the moso bamboo population. These genetic variations in different genes and alleles contribute to the flexibility and survival of moso bamboo populations in the face of changing environmental circumstances^42^. The availability of a comprehensive moso bamboo genome variation map has played an essential role in identifying possible connections between SNPs and agronomic traits. The next challenge will be to examine the associations of genetic variation with property-related phenotypes measured under various environmental conditions in the field and laboratory. This will guide and accelerate bamboo breeding on the basis of identifying genetic variations that will be useful in breeding efforts and future sustainable forests.

## Methods

### Sampling

A reasonable sampling strategy is critical for obtaining reliable results in population genetic research^43^, especially for the moso bamboo population, whose individuals have a long life cycle, strong nutritional and reproductive capacity as well as strongly affected by human activities. Additionally, the old and new individuals can be connected through a unique rhizome-dependent clonal multiplication system. First, to avoid repeated sampling, we sampled one single moso bamboo individual from nonconsecutive pieces of bamboo forest, or one dividual at a distance of more than 1 km. In this way, we sampled approximately 23–30 individuals from each bamboo forest. Second, for a population study, we need to sample natural individuals rather than cultivated individuals, while the natural distribution of moso bamboo may have been affected by human activities. Considering the difficulty of distinguishing natural and cultivated moso bamboo forests, we determined the natural forests based on consultations with local forestry authorities or bamboo farmers as mentioned before^9^. More importantly, we sampled based on the previous phylogenetic tree^9^ to improve the accuracy and efficiency of sampling. We documented the locations of each individual sampled. We obtained young leaves, quickly dried them in silica gel, and sealed them in plastic bags.

Following our sampling procedure, we sampled 427 individuals from 15 moso bamboo geographic distribution areas, representing almost all of the moso bamboo habitats in China. *Phyllostachys kwangsiensis* which was closely related to moso bamboo^44^, was selected as an outgroup. The specimens of representative individuals from different habitats were deposited at the International Centre for Bamboo and Rattan, and detailed sampling information was provided in Supplementary Table 1 and Supplementary Data 1.

### DNA extraction and sequencing

Young leaves at the vegetative growth stage were collected in August 2015 and August 2016, respectively. Total genomic DNA was extracted with the cetyltrimethylammonium bromide method^45^. The libraries of insert sizes of ~450 base-pair (bp) were constructed from randomly fragmented genomic DNA, and 150 bp paired-end reads were produced using the Illumina sequencing platform. The raw sequencing data were filtered using SOAPnuke (version 2.1.5)^46^ with parameters “-J -l 10 -q 0.1 -n 0.05”. In detail, we filtered out low-quality reads (if more than 10% of the bases had quality score lower than 10) and poly-Ns (if there were more than 5% of the based to be Ns). Meanwhile, we removed any low-quality bases (*Q* ≤ 13) or adapters at both ends. After data filtering, we used the clean data for subsequent analyses.

### SNP and InDel calling

The filtered whole-genome resequencing reads were aligned to the latest moso bamboo chromosome-level reference genome^10^ using BWA^47^ (version 0.7.12-r1039) with the parameter “-M”. We then used SAMtools^48^ (version 1.3.1) to sort alignment (BAM files), used Picard^49^ (version 1.105) to remove duplicates, and used GATK^50^ (version 3.8-1-0-g15c1c3ef) to re-align the reads around InDels.

SNP and InDel calling was performed using GATK^50^ (version 3.8-1-0-g15c1c3ef) using the joint calling method. In detail, we first obtained the genomic variant call format (GVCF) in ERC mode for each sample based on reads with mapping quality higher than 20 (using the parameters “-T HaplotypeCaller -ERC GVCF -variant_index_type LINEAR -variant_index_parameter 128000 -mq 20”), and then carried out the joint variant calling using the tool “CombineGVCFs” in GATK. In this step, low-quality mapping reads were removed (using the parameter of “-mq 20”) and we found 81.84% of the multiple mapping reads were filtered out here (Supplementary Fig. 18). To further remove possible false-positive SNPs due to multiple mapping, we identified the regions with the ratio of multiple mapping reads higher than 25% (Supplementary Fig. 19) and removed the SNPs within these regions. Since there are no available genome variation databases for VQSR in GATK, we filtered SNPs directly based on quality (filtered out if quality score lower than 50 based on quality score distribution, Supplementary Fig. 20). Finally, we excluded probably artificial SNPs by sequencing batch effect using chi-square test by PLINK^51^ (version 1.90) with parameter “-assoc”.

### SV and CNV detection and the related gene analysis

For SV identification, we used Manta^52^ (version 1.6.0) to detect insertion (INS), deletion (DEL), and inversion (INV), and BreakDancer^53^ (version 1.1.2) to detect intra-chromosome translocation (ITX) based on individual sequencing data with default parameters (Supplementary Fig. 1). Then we used SUVOVOR^54^ (version 1.0.7) to merge individual SVs using the parameter “1000 1 1 1 0 50” to allow a maximum distance of 1 kb and no more than 50% difference in length. To reduce possible false-positive SVs, we further removed the variations overlapped with tandem repeats and ambiguous bases (Ns). Finally, we only retained variation with a frequency higher than 1% using VCFtools^55^ (version 0.1.17) with the parameters “-maf 0.01 -max-maf 0.99”. We detected genes affected by SVs by extracting genes overlapped with SVs using the in-house script (available in GitHub: https://github.com/BGI-Qingdao/moso_bamboo_resequencing). Gene Ontology (GO) and KEGG pathway enrichment analyses of these genes were performed using EnrichmentPipeline (https://sourceforge.net/projects/enrichmentpipeline/, version 1.01), and a Benjamini–Hochberg corrected *p*-value < 0.05 was considered significant.

For CNV detection, we first calculated the GC-content profile in 1 kb bins along the genome and then detected CNVs using Control-FREEC^56^ (version 11.5) with default parameters. We filtered out the CNVs overlapped with ambiguous regions (Ns) and with frequencies in the population to be lower than 1%. We extracted the genes overlapped with CNVs using the in-house script (available in GitHub: https://github.com/BGI-Qingdao/moso_bamboo_resequencing) and carried out gene function enrichment using the same method described above.

### Analysis of the genome-wide heterozygous genotype frequency

To depict the genome-wide pattern of the genomic heterozygosity, we calculated two statistics to reflect the heterozygosity level of genotypes at the single-site and window levels. The single-site-level heterozygosity frequency was defined as the number of heterozygous genotypes divided by the total number of called genotypes at each site. And the window-level heterozygous genotype frequency was the average of the site-level heterozygous genotype frequency across all sites in this window. We used an in-house script (available in GitHub: https://github.com/BGI-Qingdao/moso_bamboo_resequencing) to calculate the window-level heterozygous genotype frequency in non-overlapping sliding windows of 200 kb in length. The average 200-kb-window-level heterozygous genotype frequency was 37.82%, and the standard deviation was 20.12%. Therefore, one standard deviation above the mean (57.94%) and below the mean (17.70%) was used as thresholds to distinguish high-frequency and low-frequency heterozygous regions, respectively. To avoid false-positive discovery caused by random fluctuations, high-frequency and low-frequency heterozygous regions with a continuous length greater than 5 Mb were considered as long continuous high-frequency heterozygous regions (high-LCHRs) and low-frequency heterozygous regions (low-LCHRs). We obtained genes that overlapped with high-LCHRs and low-LCHRs and the gene enrichment was performed using the method described above.

### Detection of natural selection

We applied several software to detect possible regions under selection in the moso bamboo population. First, we calculated the composite likelihood rate (CLR) and derived allele frequency (DAF) to detect possible regions under positive selection. The alleles with the status of homozygous genotypes in *Phyllostachys kwangsiensis* were considered ancestral alleles. We used SweeD^24^ (version 4.0.0) to calculate the CLR for windows of 100 kb in length (setting the parameter of “-grid”) with default parameters. We calculated DAF^25^ using an in-house script (available in https://github.com/BGI-Qingdao/moso_bamboo_resequencing) in 100-kb windows along the genome. The regions with the top 1% CLR (24.32) and DAF (0.52) were determined as potentially positively selected regions. We then detected the balancing selection based on the *B*_2_ statistics using BalLeRMix^26^ (version 2.2) with default parameters, and we determined the regions with the top 0.5% *B*_*2*_ values (270.88) to be potentially under balancing selection. We extracted the genes overlapped with these potential regions under selection as genes potentially under balancing selection. The gene enrichment was performed using the mentioned method above.

### Phylogeny construction and population structure analysis

For all individuals, we first used PLINK^51^ (version 1.90) with the parameters “-indep-pairphase 100 10 0.2” to determine a pruned SNP set to be used in the population structure analysis. In this way, we used 1,432,873 SNPs for the phylogeny and population analysis. The Identity-By-State (IBS) genetic distance matrix was calculated to quantify the correlation between individuals using the “-distance 1-ibs flat-missing” parameter in PLINK. A neighbor-joining (NJ) phylogenetic tree was constructed using the “neighbor” parameter in PHYLIP^57^ (version 3.6) based on the distance matrix. *Phyllostachys kwangsiensis* was used as the outgroup to root the phylogenetic tree. For the principal component analysis (PCA), we used EIGENSOFT^58^ (version 7.2.1) with default parameters to extract the top 10 principal components (PCs). The top three PCs were plotted using package ggplot2^59^ (version 3.3.0) in R (version 3.5.0). Maximum likelihood estimation of individual ancestries was performed using ADMIXTURE^60^ (version 1.3.0) with the parameters “-cv -j4” for multiple repeats with different random seeds. The results were visualized using the online tool CLUMPAK^61^ (accessed in July 2020).

We used the web tool Evolview^62^ (version 3) to color the phylogenetic tree. We defined five phylogenetic groups according to the phylogenetic tree. We calculated the genetic diversity (*θ*π and *θ*w) using VCFtools^55^ (version 0.1.17) with the parameters “-window-pi 100000”. We used VCFtools to calculate the population differentiation statistics (*F*_ST_) of different phylogenetic groups in 100-kb windows. We used VCFtools to calculate Tajima’s *D* in 100-kb non-overlapping windows with parameter “-TajimaD 100000”.

For the isolation-by-distance analysis, the matrix of *F*_ST_ [(*F*_ST_/(1 − *F*_ST_))] and the matrix of geographic distance among the 13 geographic distribution areas (excluding HZP, CS) were used for performing the Mantel test using ade4^63^ (version 1.7–17) package with 9999 permutations. We calculated the genetic Hamming distance matrix to quantify the relationship between individuals using the “-distance flat-missing” parameter using PLINK^51^ (version 1.90). The lowest 0.5% were visualized using Cytoscape^64^ (version 3.70).

### Inference of population demographic history

For the demographic analysis, we used PSMC^31^ (version 0.6.5-r67) and SMC++^33^ (version 1.15.4). For PSMC, we used representative individuals from the five phylogenetic groups with high sequencing depth (higher than 19×, listed in Supplementary Table 8). For SMC++, we selected more representative individuals from the five phylogenetic groups (listed in Supplementary Table 8). For SMC++, we used SNPable (http://lh3lh3.users.sourceforge.net/snpable.shtml, accessed Nov. 2019), MSMC mappability, and BEDTools^65^ (version 2.28.0) to prepare the input file (-mask, the loci to be excluded) for SMC++. For both methods, we used an estimated mutation rate of 8.51 × 10^−8^ and the generation time of 67 years^66^. The nucleotide mutation rate (*μ*) was estimated following Eq. 1

1 $${{{{{\rm{\mu }}}}}}=D\times g/2{{T}}$$

where *D* is the observed frequency of pairwise differences between two species, *T* is the estimated divergence time, and *g* is the estimated generation time for the two species^67^. *Olyra latifolia* was selected as the comparison species. In this study, we first aligned the two genomes using NUCmer^68^ (version 4.0.0), and the median of the sequence divergence was 0.1069 (Supplementary Fig. 21). The generation time (*g*) was set to 67 years^66^, and the estimated divergence time was 42.1 million years ago^69^, so that a mutation rate of 8.51 × 10^−8^ mutations per site per generation was estimated.

### Measurement of agricultural traits

We investigated nine agricultural traits of the sequenced individuals, including three growth-related traits (clear culm height, node number, and ground diameter) and six property-related traits (density, compressive strength, bending strength 12°, elastic modulus, maximum load, and tensile modulus). We measured the growth-related and property-related phenotypes according to the standards for testing the physical and mechanical properties of bamboos^70,71^. For all measurements of the quantitative traits, we adopted the mean values of three independent measurements. In addition, to control the potential impact of the environmental factors on the traits, 12 environmental factors, including latitude, longitude, altitude, soil type, annual mean precipitation, annual mean temperature, nitrogen (N), phosphorus (P), potassium (K), magnesium (Mg), boron (B), and zinc (Zn) were collected from a previous study^72^. All phenotypic measurements were provided in Supplementary Data 14.

### Genome-wide association study

For the genome-wide association study, we first removed individuals with high genetic correlations according to the pairwise IBS genetic distance (with the cutoff of 0.06), thus we remained 190 samples for the genome-wide association study. Furthermore, we used the common SNPs (minor allele frequency, MAF > 0.05, genotype missing rate <0.2). In order to minimize possible effects of environmental factors, we carried out PCA on the twelve environmental factors using function “princomp” in R package “stats”^73^ (version 4.0.4). In the meantime, the principal components of genetic variations were also calculated based on pruned SNPs. We used the first three PCs of environmental factors and the first three PCs (Q) of genetic variations as fixed effects to correct for stratification. A kinship (K) matrix in the emmax-bin-intel package in EMMAX (version beta-07Mar2010)^74^ was used as a random effect to model the population relatedness in GWAS.

To increase the statistical power of the association analysis, a comprehensive strategy was used based on four different methods (Supplementary Fig. 13), including the mixed linear model (EMMAX), fixed and random model circulating probability unification with high statistical power (FarmCPU) by GAPIT (version 3.0, released 2018.08.18)^75^, MAGMA-based gene-level analysis (version 1.07bb)^76^, and mrMLM-based multi-locus association (ISI EM-BLASSO) (version 4.0)^77^ using the Q + K model with default parameters. To calculate the *p*-value threshold, we firstly estimated the effective SNP number using the genetic type I error calculator (GEC)^78^ (version 0.2) with default parameters. The *p*-value threshold was 2.10 × 10^−8^ for EMMAX and FarmCPU by Bonferroni correction method (that is, 0.05/the number of effective SNPs estimated by GEC (2,384,433 SNPs, Supplementary Table 9)), and the *p*-value threshold of 1.08 × 10^−6^ for MAGMA was also calculated by Bonferroni correction (that is, 0.05/total number of genes with SNPs (46,088 genes)). LOD (log of odds) value > 3 is a suggestive significance threshold by ISI EM-BLASSO^77^.

Finally, we used an integrated approach of combining the above results to depict more candidate genes with sight genetic effect. In detail, we first picked up the SNPs above significant *p*-value threshold as candidate SNPs, and SNPs in the top 500 significance in each method and meanwhile identified by more than two methods were also considered as candidate SNPs. We further found the MTAs (marker-trait associations) by merge these candidate SNPs within 10 kb distance. The genes within or the 5 kb upstream or downstream from the MTAs were considered as candidate genes. In addition, the related co-expression networks of moso bamboo were generated and visualized via the BambooNET website^79^ (http://bioinformatics.cau.edu.cn/bamboo/, accessed May 1, 2021).

### Reporting summary

Further information on research design is available in the Nature Research Reporting Summary linked to this article.

## Supplementary information

Supplementary Information

Supplementary Data

Description of Additional Supplementary Files

Reporting Summary

## Data Availability

The data supporting the findings of this work are available within the paper and its Supplementary Information files. A reporting summary for this article is available as a Supplementary Information file. The sequencing data in this study have been deposited in the NCBI Sequence Read Archive under accession number PRJNA755164 or the China National GeneBank (CNGB) under accession number CNP0001535. The datasets of twelve environmental factors were adapted from a previous study^72^. ETOPO2v2c Global Gridded 2-min elevation and bathymetric data are available at US National Centers for Environmental Information library (10.7289/V5J1012Q; https://www.ncei.noaa.gov/access/metadata/landing-page/bin/iso?id=gov.noaa.ngdc.mgg.dem:301). Source data are provided with this paper.

## Source data

source data

## Code availability

Customized codes used in this study have been deposited in GitHub [https://github.com/BGI-Qingdao/moso_bamboo_resequencing] and are also publicly available in the Zenodo repository [10.5281/zenodo.5201511].
