# Supplementary material for: Analysis of 427 genomes reveals moso bamboo population structure and genetic basis of property traits
Source: Nat Commun. 2021 Sep 15;12:5466. doi: 10.1038/s41467-021-25795-x (PMC8443721; doi:10.1038/s41467-021-25795-x)
Supplement: Supplementary file 1 — Supplementary Information [file 41467_2021_25795_MOESM1_ESM.pdf]

Analysis of 427 genomes reveals moso bamboo population structure and genetic basis of property traits

Zhao *et al.*

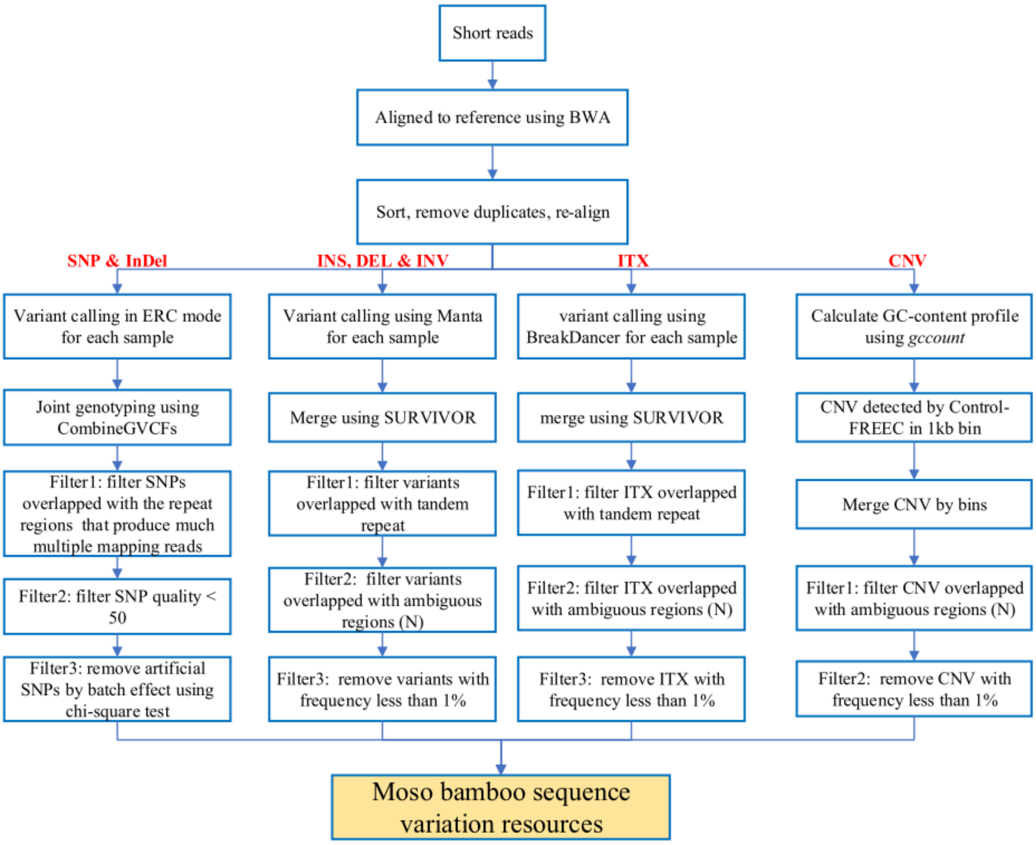

Supplementary Fig. 1. The flowchart of detecting different types of variation, including SNP, small InDel, INS, INV, ITX and CNV. SNP, single nucleotide polymorphism; InDel, small insertion and deletion (common < 50 bp); INS, insertion; INV, inversion; ITX: intra-chromosome translocation; CNV: copy number variation.

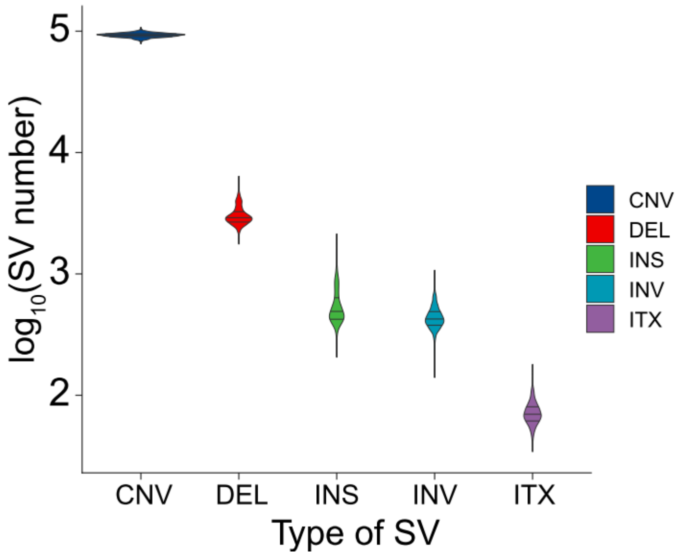

Supplementary Fig. 2. Violin plot of the distribution of SVs and CNVs of different individuals. The line in middle represents the median value and the upper and lower lines represent the 1st and 3rd quartile values. Source data are provided as a Source Data file.

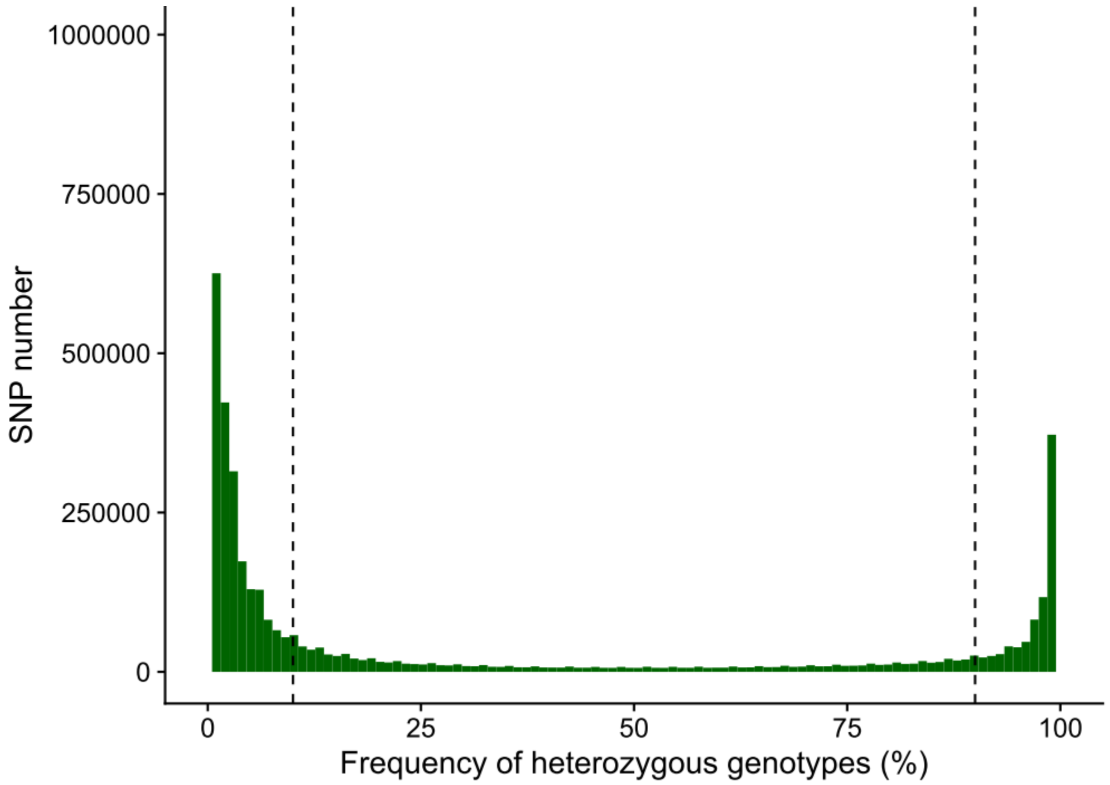

Supplementary Fig. 3. Frequency distribution histogram of the frequency of heterozygous genotypes. The left dashed line represents the cutoff of 10%, which means that the no more than 10% of individuals with heterozygous genotypes in sequenced individuals were indicated as low-frequency heterozygous genotypes. There are 1,776,063 SNPs with low-frequency heterozygous genotypes, which account for 34.55% of total SNPs. The right dashed line represents the cutoff of 90%, which means that the more than 90% of individuals with heterozygous genotypes in sequenced individuals were indicated as high-frequency heterozygous genotypes. There are 2,385,795 SNPs with low-frequency heterozygous genotypes, which account for 46.42% of total SNPs. Source data are provided as a Source Data file.

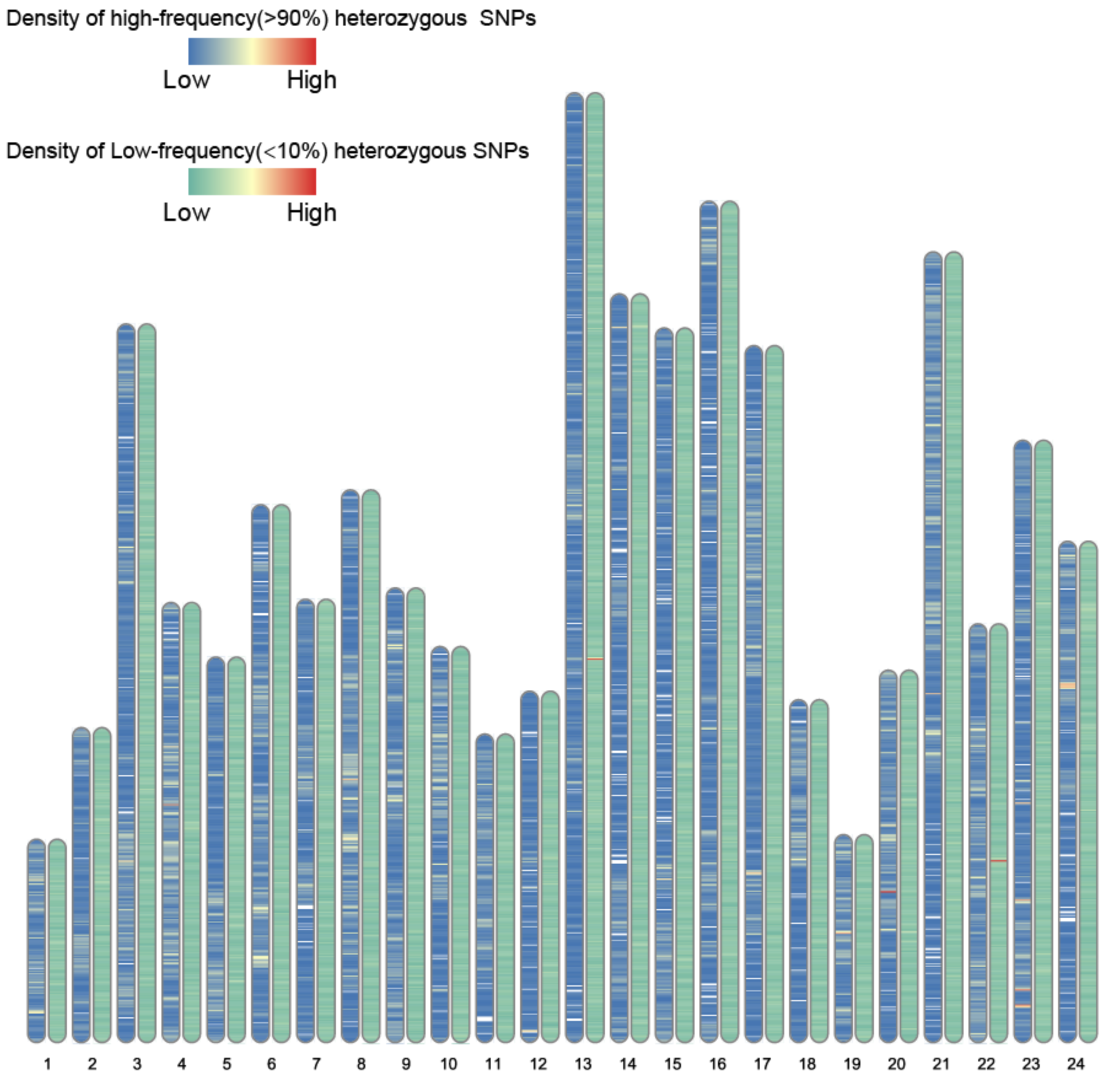

Supplementary Fig. 4. The density of two types of heterozygous sites on chromosomes within non-overlapping windows of 200 kb length. Low-frequency heterozygous SNPs: the individuals with heterozygous genotypes no more than 10% in moso bamboo population, and high-frequency heterozygous SNPs: the individuals with heterozygous genotype no less than 90% in moso bamboo population. Source data are provided as a Source Data file.

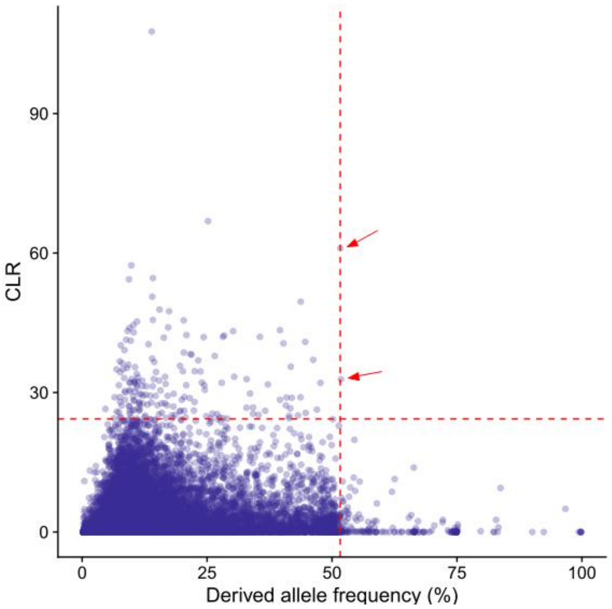

Supplementary Fig. 5. The positive selection detected using CLR and DAF. The non-overlapping windows of 100 kb length were used and the red arrows represent the identified positive selection regions, and however the signatures were overall not notable. Source data are provided as a Source Data file.

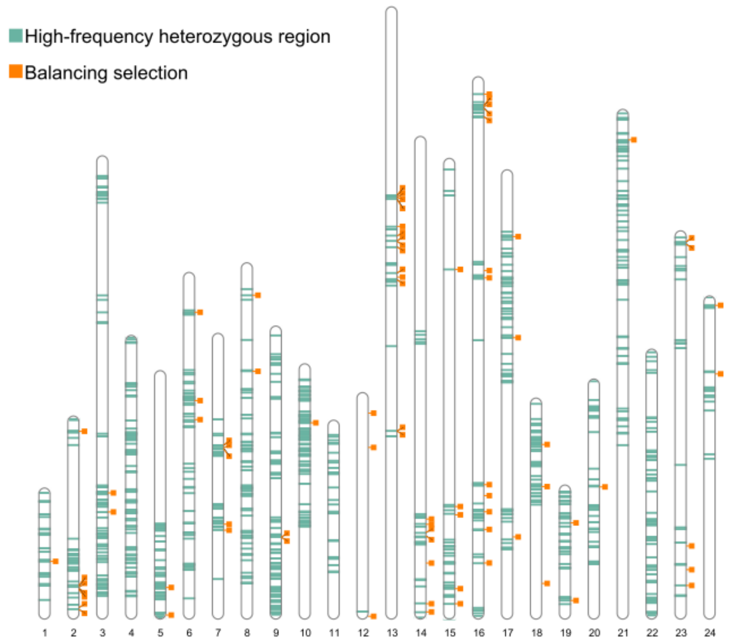

Supplementary Fig. 6. The distribution of high-frequency heterozygous regions and balancing selection regions on chromosomes. The high-frequency heterozygous regions were calculated in non-overlapping windows of 200 Kb length, and the top 10% heterozygous ratio were considered as high-frequency heterozygous regions. Source data are provided as a Source Data file.

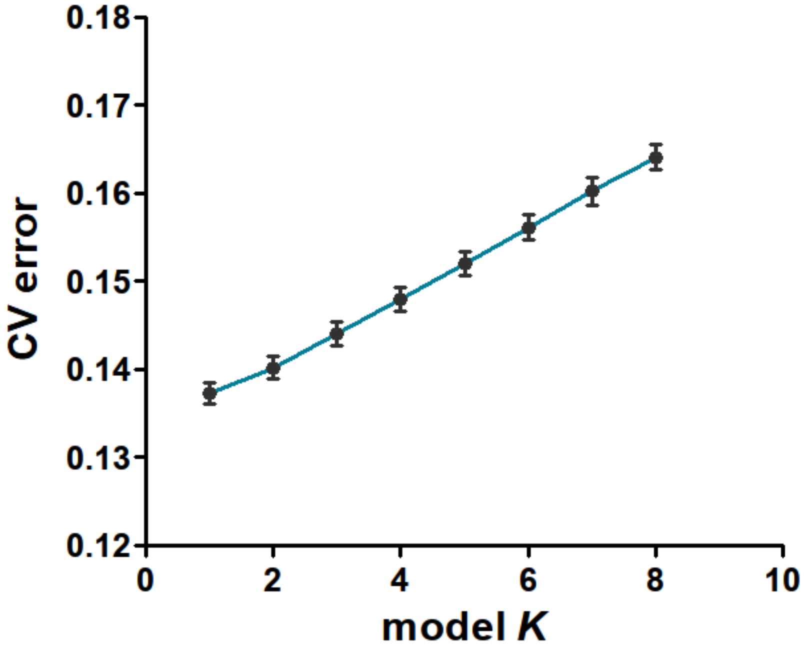

Supplementary Fig. 7. The CV error value for each model *K*. Ten repeats with different random seed for each different model *K* from 1 to 8 were tested. The black points represent the mean values and the upper and lower lines for 95% CI (confidence interval). Source data are provided as a Source Data file.

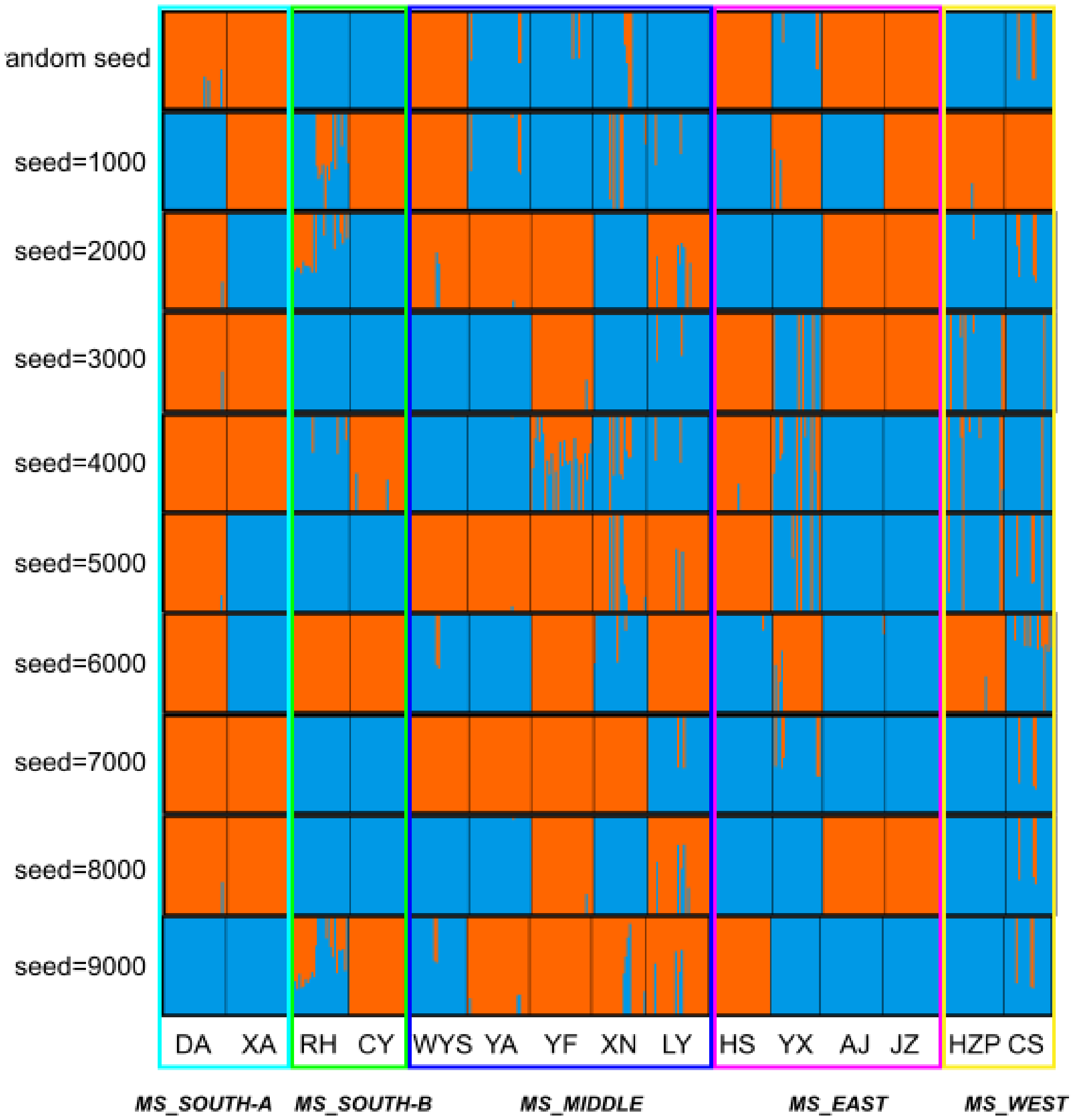

Supplementary Fig. 8. The admixture results (model *K*=2) for the moso bamboo population. Source data are provided as a Source Data file.

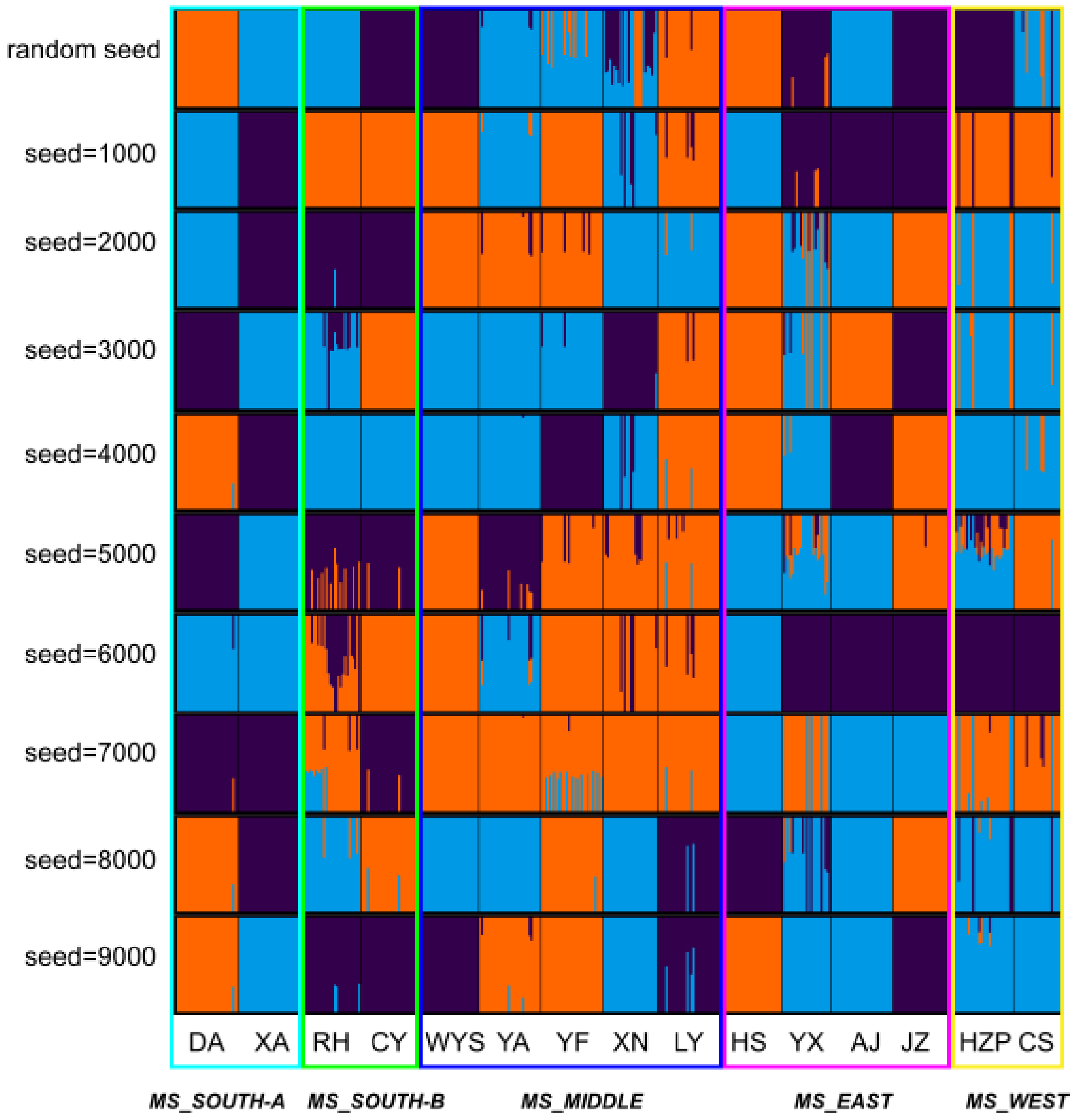

Supplementary Fig. 9. The admixture results (model *K*=3) for the moso bamboo population. Source data are provided as a Source Data file.

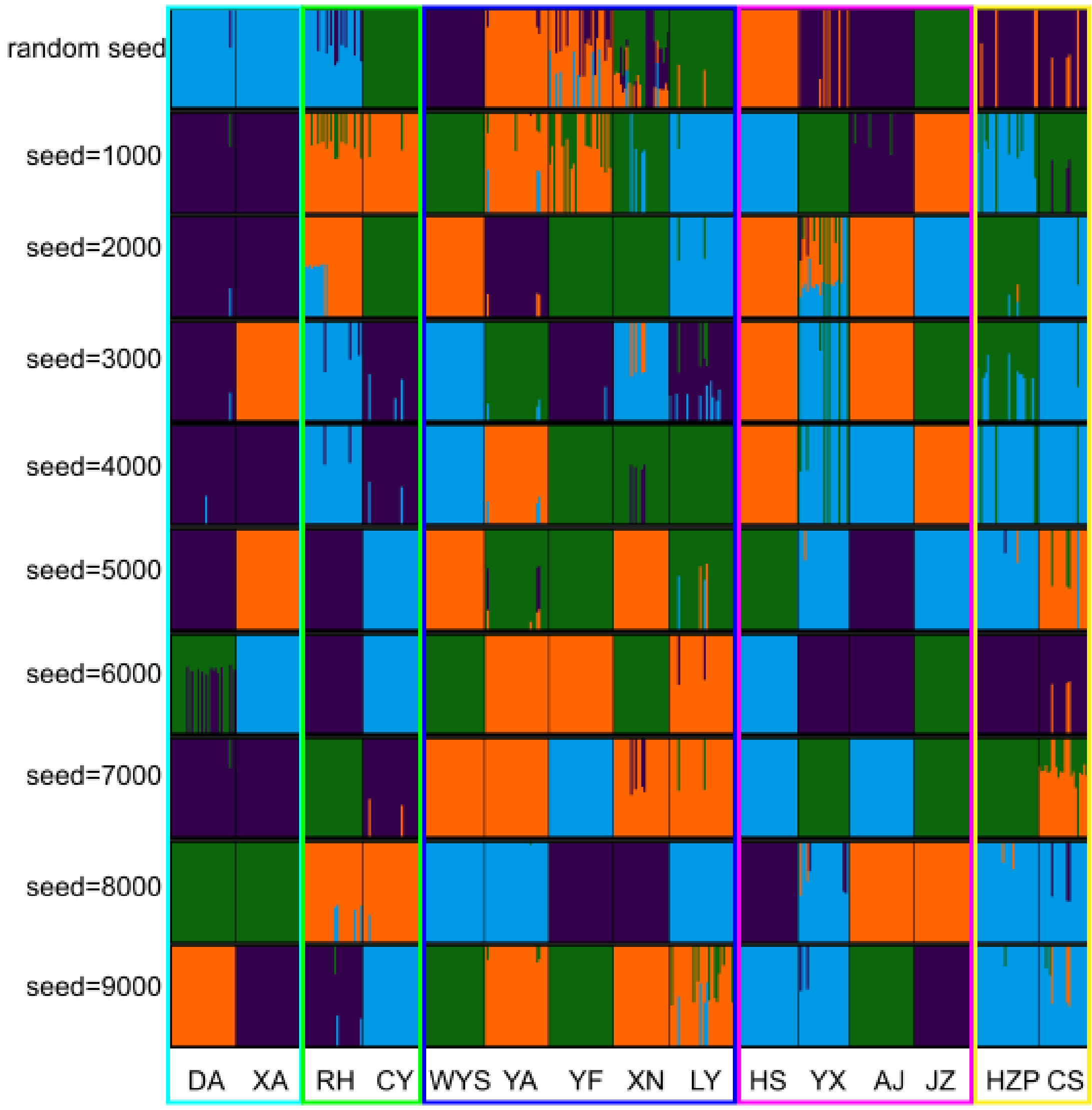

Supplementary Fig. 10. The admixture results (model *K*=4) for the moso bamboo population. Source data are provided as a Source Data file.

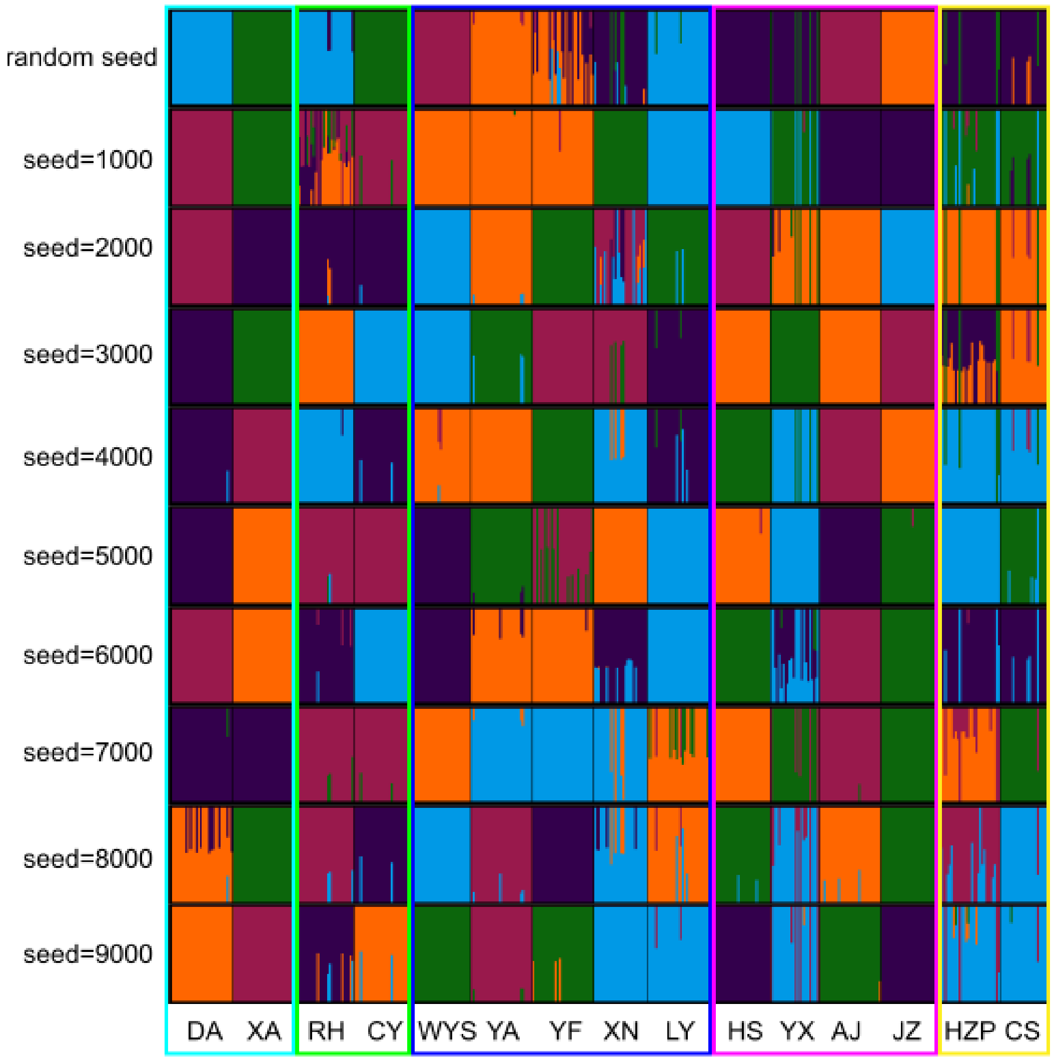

Supplementary Fig. 11. The admixture results (model *K*=5) for the moso bamboo population. Source data are provided as a Source Data file.

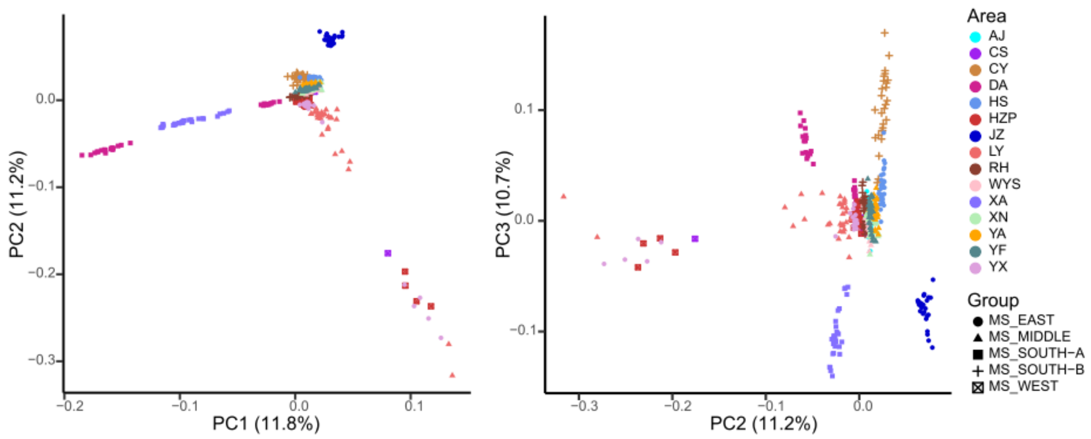

Supplementary Fig. 12. The scatter plot of the 1st, 2nd, and 3rd principal components (PCs) in the PC analysis of the moso bamboo population using smartPCA. Source data are provided as a Source Data file.

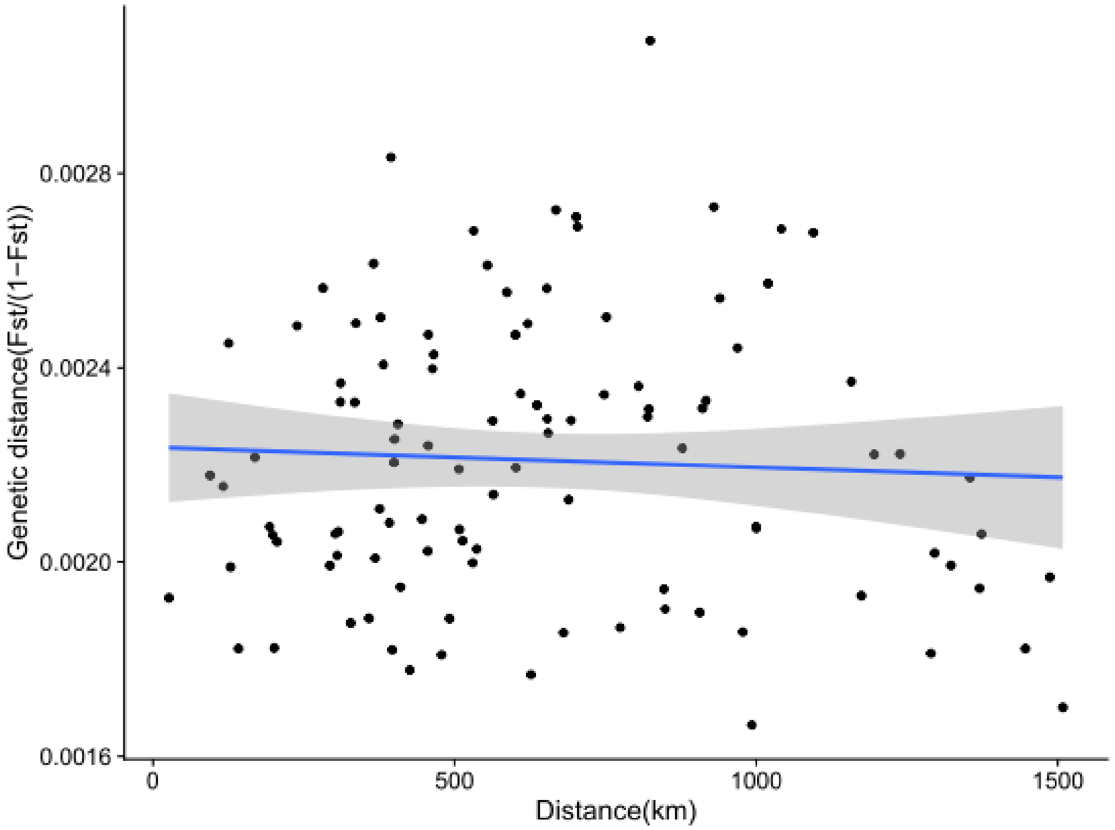

Supplementary Fig. 13. Mantel test to examine the relationship between geographic distance and genetic distance for 15 geographic areas. The blue line was fitted by the linear regression between genetic distance and geographic distance on the basis of ordinary least squares in the function "geom_smooth" from ggplot2. No significant correlation was observed (*p*-value = 0.5723, r = -0.0512) by the Mantel test using ade4 package, and the *p*-value was calculated using a one-sided Mantel test with 9999 permutations. The gray error band represents the 95% CI (confidence interval). Source data are provided as a Source Data file.

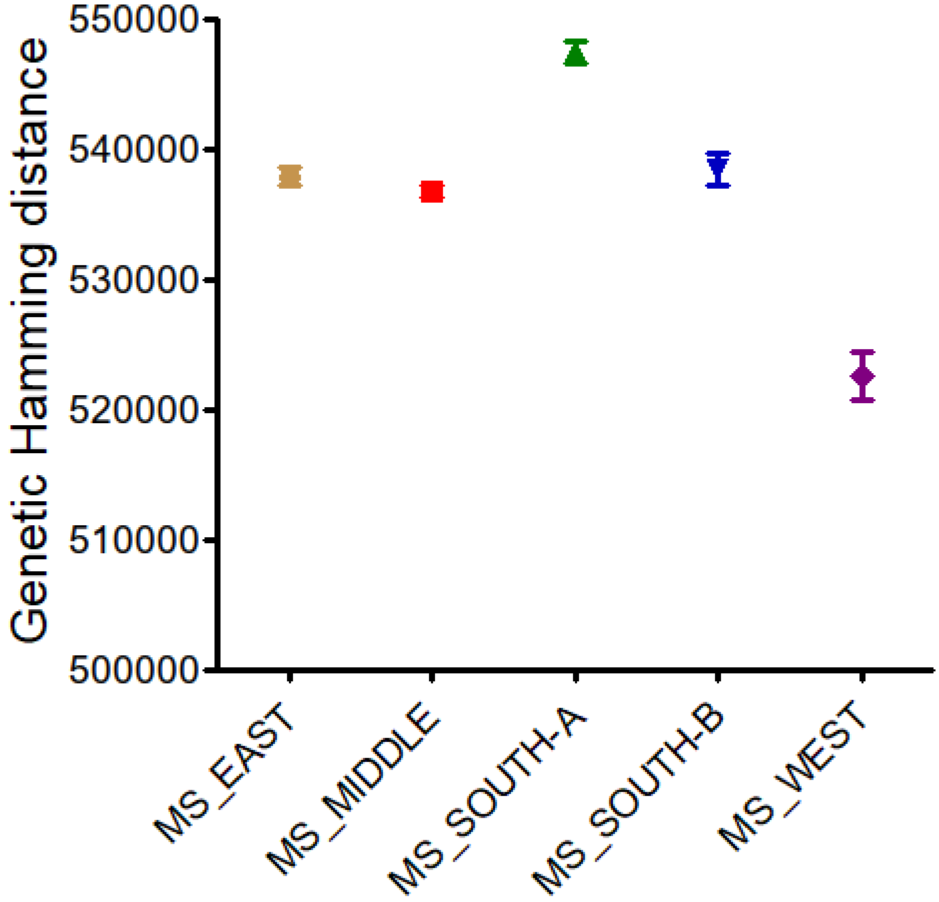

Supplementary Fig. 14. The distribution of intra-phylogenetic pairwise genetic distance (Hamming distance) between accessions of each group. The numbers of calculated values of pairwise genetic distance are 6216, 10585, 1770, 1540 and 378 for MS_EAST, MS_MIDDLE, MS_SOUTH-A, MS_SOUTH-B, and MS_WEST, respectively. The center point represents the mean value and the upper and lower lines for 95% CI (confidence interval). Source data are provided as a Source Data file.

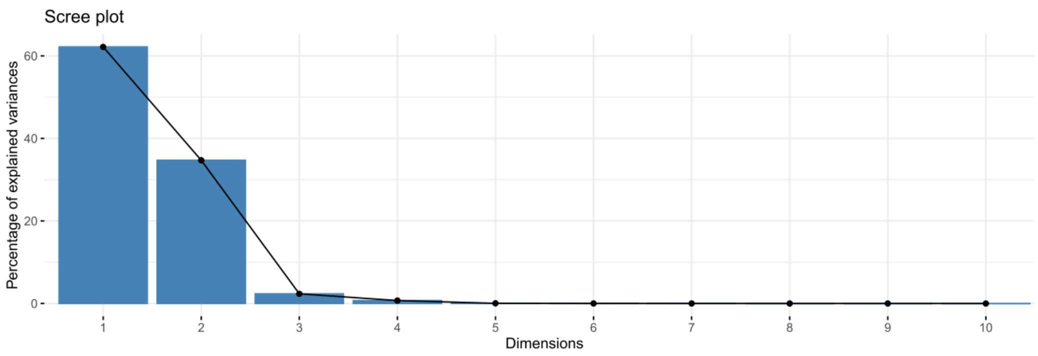

Supplementary Fig. 15. The screen plot showed the percentage of explained variances along with the increase of dimensions. Source data are provided as a Source Data file.

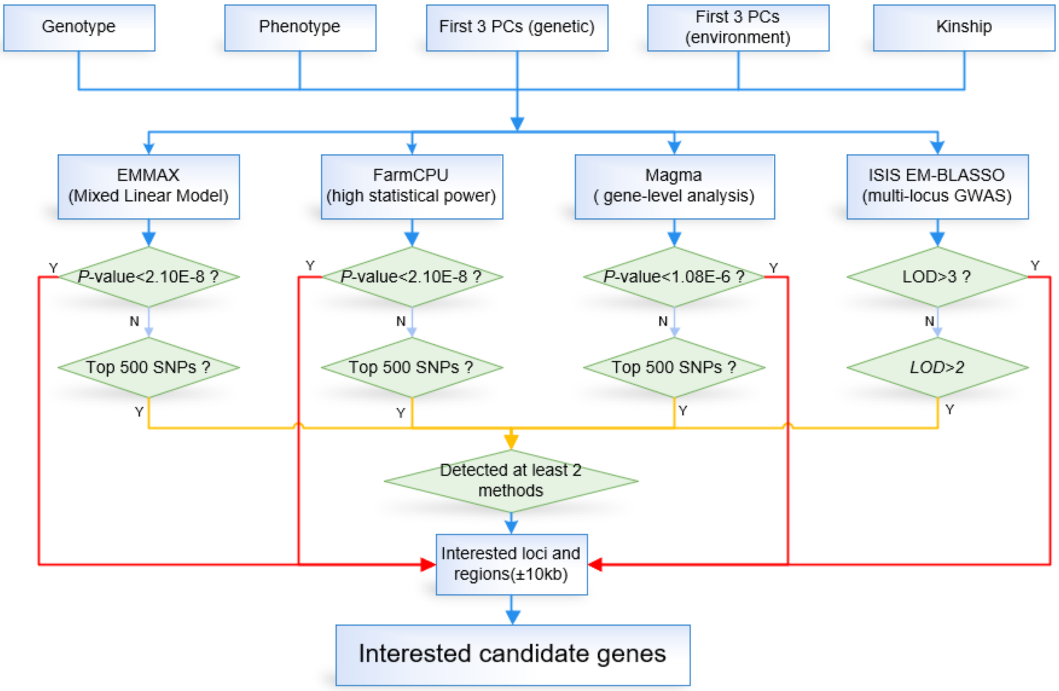

Supplementary Fig. 16. The pipeline of detecting the marker-trait associations (MTAs) based on four different GWAS methods. The red lines represent "threshold_of_BH_adjusted_Pvalue", that means exceeding the threshold of Benjamini-Hochberg correction *p*-value. The yellow lines represent "intersection_of_top_500_SNPs", that is supported by top 500 significant SNPs of two different GWAS methods. The details of GWAS results can be found in Supplementary Data 16-24 for nine traits separately.

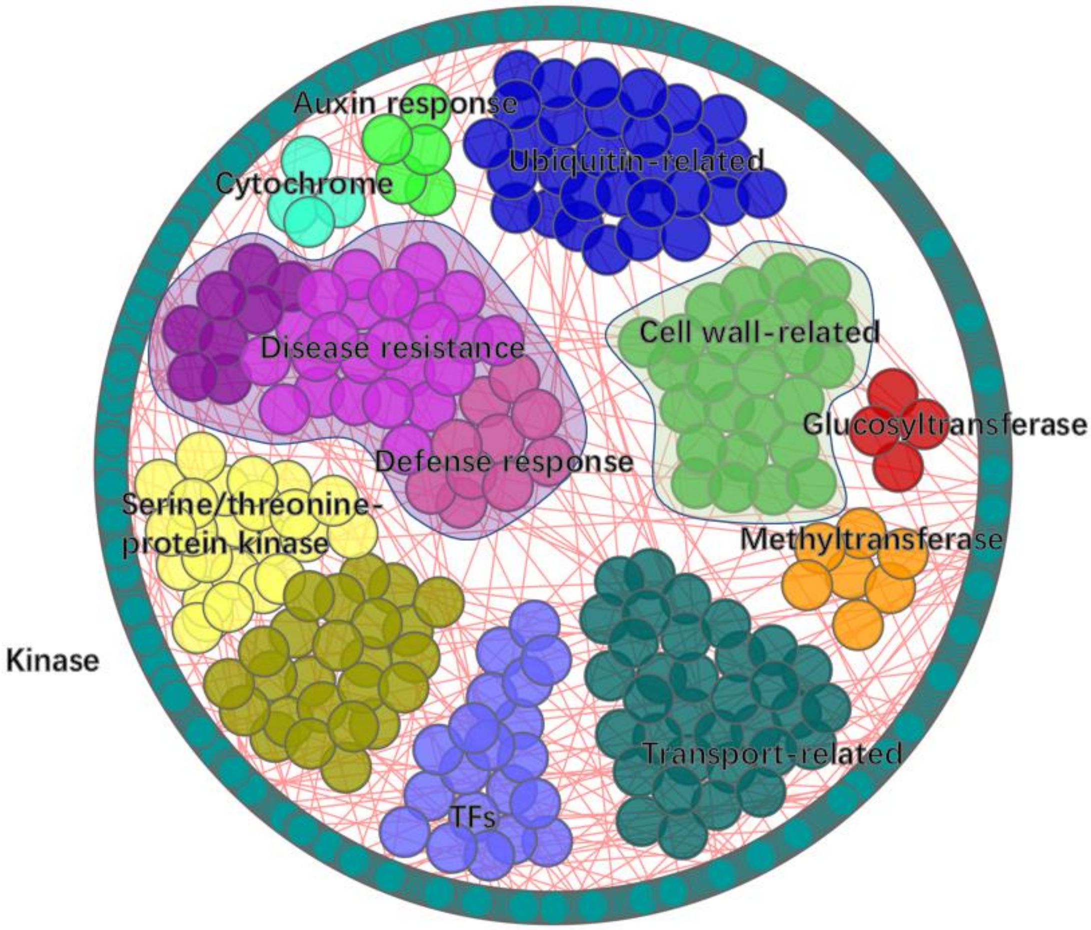

Supplementary Fig. 17. Co-expression network for candidate genes identified by GWAS. Source data are provided as a Source Data file.

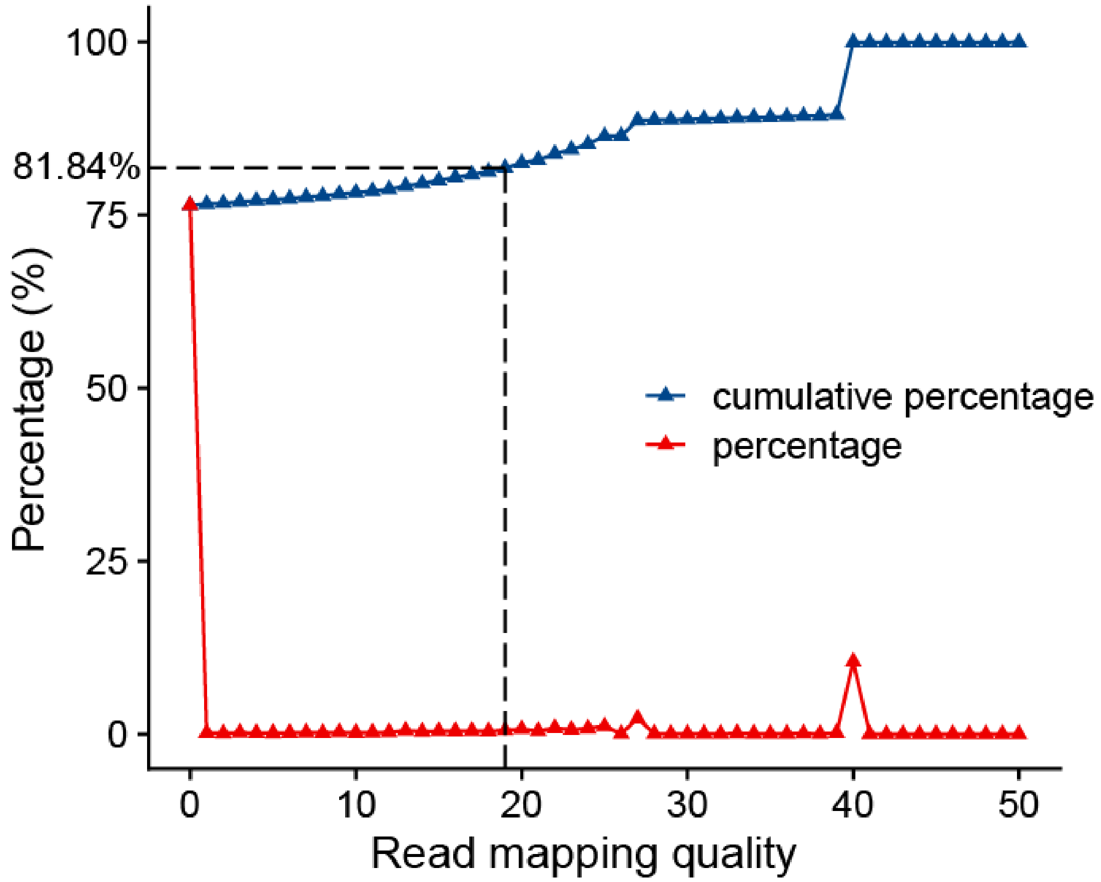

Supplementary Fig. 18. The distribution of mapping quality of multi-mapping reads. Most multi-mapping reads were with low mapping quality. In the process of SNP and InDel calling using GATK, only mapping quality >= 20 was used, which means the 81.84% of multi-mapping reads were removed before SNP and InDel calling (showed by dashed lines on x-axis and y-axis). Source data are provided as a Source Data file.

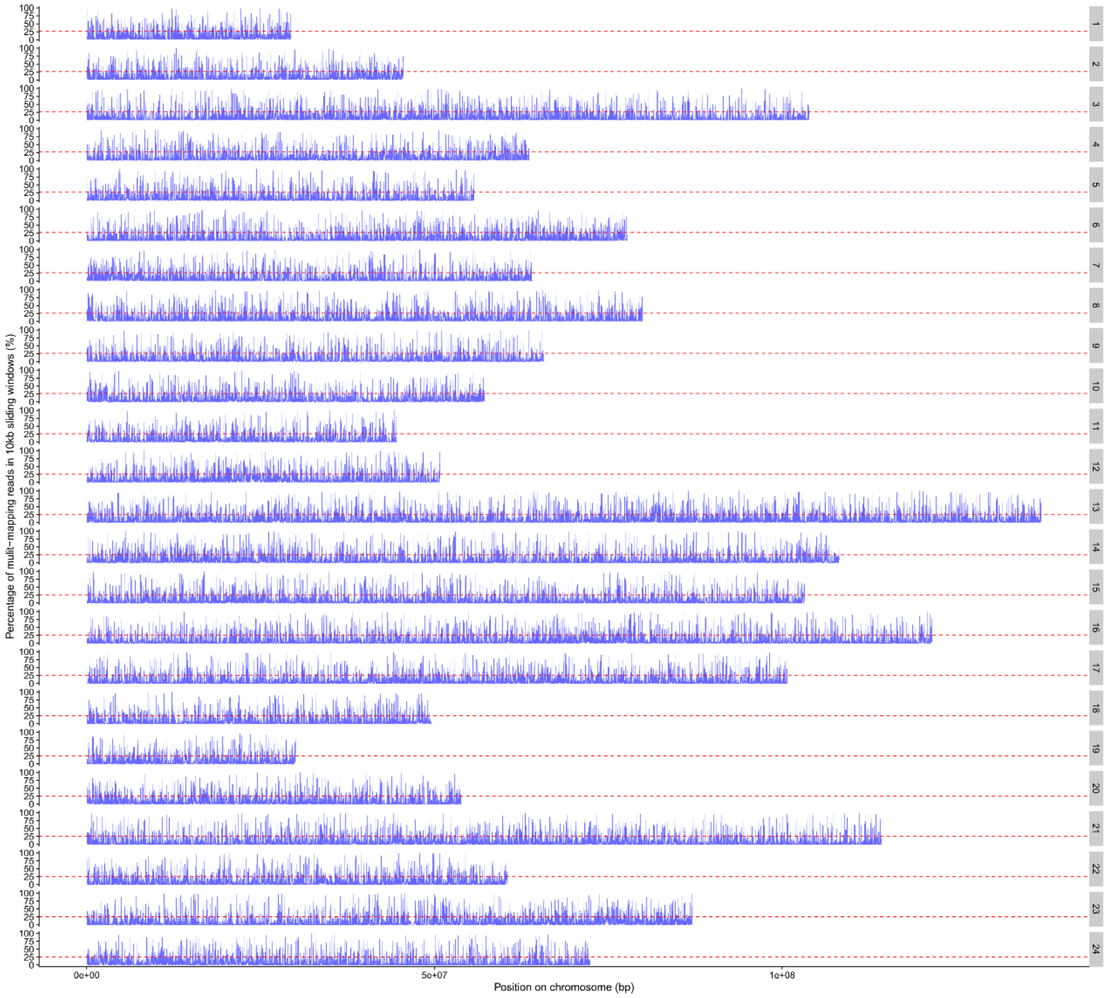

Supplementary Fig. 19. The genome-wide distribution of the percentage of multi-mapping reads on the moso bamboo reference. The regions producing the percentage of multiple mapping reads > 25% were identified as tricky regions trending to lead to false positive, and the SNPs on these regions were removed. The red dashed lines showed the cutoff of 25%. Source data are provided as a Source Data file.

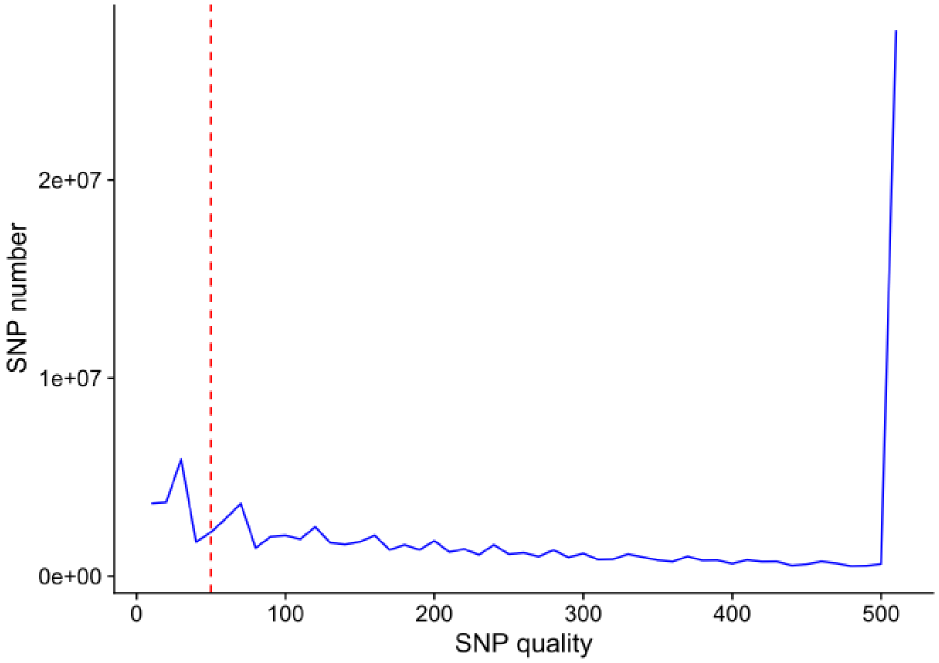

Supplementary Fig. 20. The distribution of the quality of SNPs. The cutoff of 50 was used to remove low-quality SNPs as showed in red dashed line. Source data are provided as a Source Data file.

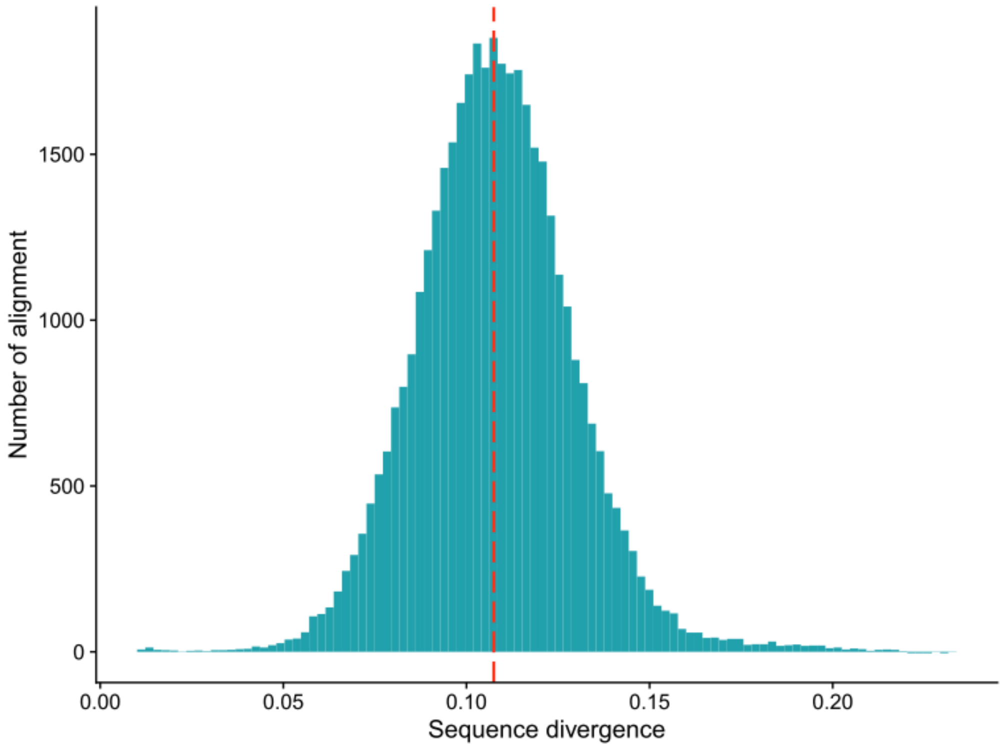

Supplementary Fig. 21. The distribution of sequence divergence between moso bamboo and *Olyra latifolia*. The sequence divergence was calculated for each alignment block excluding gaps, which was produced by genome-genome alignment using NUCmer. The median value of sequence divergence is 0.1069 as showed by red dashed line. Source data are provided as a Source Data file.

Supplementary Table 1. The details of 427 representative moso bamboo samples from 15 geographic areas of China.

Areas	Abbreviation of areas	Number of samples	Assigned groups	Group size
Yong'an, Fujian	YA	30	MS_MIDDLE	146
Wuyishan, Fujian	WYS	30		
Longyou, Zhejiang	LY	30		
Xianning, Hubei	XN	26		
Yifeng, Jiangxi	YF	30		
Dong'an, Hunan	DA	30	MS_SOUTH-A	60
Xing'an, Guangxi	XA	30		
Renhua, Guangdong	RH	29	MS_SOUTH-B	56
Chongyi, Jiangxi	CY	27		
Huangshan, Anhui	HS	30	MS_EAST	112
Jinzhai, Anhui	JZ	28		
Yixing, Jiangsu	YX	24		
Anji, Zhejiang	AJ	30		
Chishui, Guizhou	CS	23	MS_WEST	53
Haiziping, Yunnan	HZP	30		
Total	--	427	--	427

Supplementary Table 2. The statistics of total and linkage disequilibrium pruned SNPs.

Chromosome	Length (bp)	SNP number	Variant's rate (bp/SNP)	SNP number (linkage disequilibrium pruned)
1	29,393,643	101,688	289.06	18,935
2	45,530,765	141,257	322.33	33,563
3	103,881,109	307,295	338.05	82,025
4	63,624,372	206,545	308.04	43,452
5	55,715,837	164,406	338.89	42,889
6	77,743,242	222,102	350.03	55,230
7	64,081,027	174,852	366.49	49,144
8	79,898,979	238,165	335.48	57,903
9	65,709,403	209,010	314.38	47,819
10	57,248,354	170,380	336.00	38,355
11	44,603,463	131,554	339.05	32,118
12	50,795,940	122,857	413.46	42,298
13	137,299,170	373,241	367.86	117,394
14	108,238,415	268,777	402.71	78,092
15	103,306,719	260,608	396.41	74,923
16	121,622,346	308,957	393.65	90,947
17	100,753,730	292,751	344.16	78,570
18	49,564,831	131,769	376.15	33,458
19	30,060,168	105,183	285.79	22,998
20	53,830,895	176,525	304.95	43,704
21	114,306,658	331,468	344.85	85,259
22	60,531,284	203,298	297.75	47,450
23	87,068,002	240,325	362.29	63,549
24	72,468,335	184,206	393.41	47,147
Unplaced	130,326,903	378,792	344.06	105,651
**Total**	**1,907,603,590**	**5,446,011**	**350.28**	**1,432,873**

Supplementary Table 3. The statistics of the detected small InDels.

Chromosome	Length (bp)	InDel number	Variant's rate (bp/InDel)
1	29,393,643	21,751	1351.37
2	45,530,765	27,864	1634.04
3	103,881,109	59,961	1732.48
4	63,624,372	38,613	1647.74
5	55,715,837	30,759	1811.37
6	77,743,242	44,875	1732.44
7	64,081,027	35,137	1823.75
8	79,898,979	45,101	1771.56
9	65,709,403	40,351	1628.45
10	57,248,354	34,984	1636.42
11	44,603,463	25,967	1717.70
12	50,795,940	24,847	2044.35
13	137,299,170	71,368	1923.82
14	108,238,415	56,824	1904.80
15	103,306,719	55,299	1868.15
16	121,622,346	62,694	1939.94
17	100,753,730	56,050	1797.57
18	49,564,831	29,470	1681.87
19	30,060,168	18,344	1638.69
20	53,830,895	32,987	1631.88
21	114,306,658	64,085	1783.67
22	60,531,284	39,025	1551.09
23	87,068,002	48,198	1806.47
24	72,468,335	38,992	1858.54
unplaced	130,326,903	74,545	1748.30
**Total**	**1,907,603,590**	**1,078,091**	**1769.43**

Supplementary Table 4. SNPs in different genomic regions.

Regions	Annotation	Putative Impact	SNP number	Percentage (%)
Intergenic	--	--	5,117,675	93.971
UTR	--	--	34	0.001
Intron	splice site donor	high	212	0.004
	splice site acceptor	high	274	0.005
	other	--	172,776	3.173
Exon	start lost	high	57	0.001
	stop gained	high	3,311	0.061
	stop lost	high	2,831	0.001
	non-synonymous coding	moderate	88,998	1.634
	synonymous coding	low	59,204	1.087
	other	--	557	0.010
Other	rare amino acid	-	82	0.002

Supplementary Table 5. The statistics of detected SVs, CNVs and affected genes.

Variation type†	Number	Percentage of SVs (%)	Affected genes
**SV**	**21,042**	**100**	**8,730 (7,483††)**
• INS	5,043	23.97	1,152
• DEL	11,631	55.28	4,318
• INV	4,063	19.30	3,074
• ITX	305	1.45	186
**CNV**	**168,700**	**100**	**3,306**

† INS: insertion, DEL: deletion, INV: inversion, ITX: intra-chromosome translocation.
†† remove the duplicated genes

Supplementary Table 6. Identified two potential regions and one candidate gene under positive selection for the moso bamboo population.

**Chr.**	6	19
**Start**	46,282,346	5,716,986
**End**	46,291,048	5,725,958
**Genes**	NA†	PH02Gene49545.t1
**Nr annotation**	NA	gi|475582649|gb|EMT19191.1|/5.89982e-146/hypothetical protein F775_13597 [*Aegilops tauschii*]
**KEGG ortholog annotation**	NA	NA
**Interpro annotation**	NA	PF04578/3.9E-24/Protein of unknown function, DUF594
**GO annotation**	NA	NA

†NA= non-available

Supplementary Table 7. The population parameters of five phylogenetic groups.

Subpopulation	Number of Accessions	SNP number	$\theta\pi$	θw	Tajima'*D*
MS_EAST	112	4,896,197	7.017E-04	4.211E-04	1.33814
MS_MIDDLE	146	5,046,064	7.004E-04	4.157E-04	1.35485
MS_SOUTH-A	60	4,466,285	7.046E-04	4.287E-04	1.37604
MS_SOUTH-B	56	4,429,689	7.057E-04	4.307E-04	1.36832
MS_WEST	53	4,392,072	7.033E-04	4.432E-04	1.32585
whole-population	427	5,446,011	6.991E-04	3.871E-04	1.62645

Supplementary Table 8. The individuals used in the inference of demographic history.

Group	ID	Sequencing depth(X)	PSMC	SMC++
MS_EAST	AJ-12	28.99	√	
MS_EAST	HS-12	26.79		√
MS_EAST	HS-15	31.08		√
MS_EAST	HS-17	25.85		√
MS_EAST	HS-22	36.36		√
MS_EAST	HS-24	30.05		√
MS_EAST	HS-25	24.04		√
MS_EAST	HS-26	28.39		√
MS_EAST	HS-28	29.96		√
MS_EAST	HS-29	27.39		√
MS_EAST	HS-30	29.62		√
MS_EAST	JZ-1	21.33		√
MS_EAST	JZ-15	20.12		√
MS_EAST	JZ-21	22.18		√
MS_EAST	JZ-3	22.05		√
MS_EAST	YX-13	23.23		√
MS_EAST	YX-14	24.74		√
MS_EAST	YX-15	24.86		√
MS_EAST	YX-16	23.45		√
MS_EAST	YX-17	26.98		√
MS_EAST	YX-19	24.16		√
MS_EAST	YX-2	22.32		√
MS_EAST	YX-20	21.76		√
MS_EAST	YX-21	20.25		√
MS_EAST	YX-22	20.06		√
MS_EAST	YX-27	22.15		√
MS_EAST	YX-29	21.38		√
MS_EAST	YX-3	21.78		√
MS_MIDDLE	LY-1	24.55		√
MS_MIDDLE	LY-17	20.6		√
MS_MIDDLE	LY-19	21.21		√
MS_MIDDLE	LY-2	20.12		√
MS_MIDDLE	LY-22	26.84		√
MS_MIDDLE	LY-27	23.06		√
MS_MIDDLE	LY-28	22.51		√
MS_MIDDLE	WYS-1	27.87		√
MS_MIDDLE	WYS-11	25.22		√
MS_MIDDLE	WYS-13	32.72		√
MS_MIDDLE	WYS-16	30.96		√
MS_MIDDLE	WYS-22	27.06		√
MS_MIDDLE	WYS-24	26.96		√

MS_MIDDLE	WYS-26	26.43		√
MS_MIDDLE	WYS-27	26.29		√
MS_MIDDLE	XN-12	24.08		√
MS_MIDDLE	XN-13	27.16		√
MS_MIDDLE	XN-14	26.54		√
MS_MIDDLE	XN-19	31.1		√
MS_MIDDLE	XN-20	22.11		√
MS_MIDDLE	XN-21	26.97		√
MS_MIDDLE	XN-27	24.63		√
MS_MIDDLE	XN-29	28.89		√
MS_MIDDLE	XN-3	33.17		√
MS_MIDDLE	XN-5	26.51		√
MS_MIDDLE	XN-6	28.46		√
MS_MIDDLE	XN-7	25.4		√
MS_MIDDLE	YA-13	29.52	√	√
MS_MIDDLE	YA-14	28.09		√
MS_MIDDLE	YA-29	27.74		√
MS_MIDDLE	YF-6	21.94		√
MS_SOUTH-A	DA-7	27.95	√	
MS_SOUTH-A	XA-2	19.57		√
MS_SOUTH-A	XA-3	20.16		√
MS_SOUTH-A	XA-5	21.66		√
MS_SOUTH-A	XA-8	20.08		√
MS_SOUTH-B	CY-15	21.35		√
MS_SOUTH-B	CY-17	21.68		√
MS_SOUTH-B	CY-19	21.29		√
MS_SOUTH-B	CY-22	21.76		√
MS_SOUTH-B	CY-25	26.02		√
MS_SOUTH-B	CY-7	21.05		√
MS_SOUTH-B	RH-15	28.24	√	√
MS_SOUTH-B	RH-18	24.5		√
MS_SOUTH-B	RH-22	24.31		√
MS_SOUTH-B	RH-28	20.58		√
MS_WEST	CS-13	29.1		√
MS_WEST	CS-18	21.36		√
MS_WEST	CS-19	20.02		√
MS_WEST	CS-20	23.01		√
MS_WEST	CS-22	22.38		√
MS_WEST	CS-25	21.58		√
MS_WEST	CS-26	22.21		√
MS_WEST	CS-3	22.95		√
MS_WEST	HZP-1	27.96	√	
MS_WEST	HZP-13	20.34		√
MS_WEST	HZP-14	25.39		√

MS_WEST	HZP-15	42.95	√
MS_WEST	HZP-28	24.93	√
MS_WEST	HZP-29	24.57	√
MS_WEST	HZP-30	21.24	√
MS_WEST	HZP-6	20.79	√

Supplementary Table 9. The statistics of SNPs used in genome-wide association study.

Chromosome	Length (bp)	SNP number	Variant's rate (bp/SNP)
1	29,393,643	64,179	457.99
2	45,530,765	78,611	579.19
3	103,881,109	162,350	639.86
4	63,624,372	130,742	486.64
5	55,715,837	89,170	624.83
6	77,743,242	124,301	625.44
7	64,081,027	87,069	735.98
8	79,898,979	136,201	586.63
9	65,709,403	125,605	523.14
10	57,248,354	100,186	571.42
11	44,603,463	74,965	594.99
12	50,795,940	49,728	1021.48
13	137,299,170	168,072	816.91
14	108,238,415	125,196	864.55
15	103,306,719	122,692	842.00
16	121,622,346	147,246	825.98
17	100,753,730	155,363	648.51
18	49,564,831	72,456	684.07
19	30,060,168	65,623	458.07
20	53,830,895	100,528	535.48
21	114,306,658	181,484	629.84
22	60,531,284	121,592	497.82
23	87,068,002	121,610	715.96
24	72,468,335	93,634	773.95
unplaced	130,326,903	216,607	601.67
Total	1,907,603,590	**2,915,210**	654.36
Effective SNP estimated by GEC[†]	1,907,603,590	**2,384,433**	800.02

[†] Effective SNP number was estimated by Genetic Type I error calculator (GEC) version 0.2.
