# Supplementary material for: Analysis of 427 genomes reveals moso bamboo population structure and genetic basis of property traits
Source: Nat Commun. 2021 Sep 15;12:5466. doi: 10.1038/s41467-021-25795-x (PMC8443721; doi:10.1038/s41467-021-25795-x)
Supplement: Supplementary file 3 — Description of Additional Supplementary Files [file 41467_2021_25795_MOESM3_ESM.pdf]

Description of Additional Supplementary Files

File Name: Supplementary Data 1

Description: The statistics of sequencing data mapping results.

File Name: Supplementary Data 2

Description: The number of SVs and CNVs of each individual.

File Name: Supplementary Data 3

Description: List of genes affected by SVs and CNVs.

File Name: Supplementary Data 4

Description: KEGG enrichment analysis for the affected genes by more than two different types of SVs.

File Name: Supplementary Data 5

Description: KEGG enrichment analysis for the affected genes overlapped with copy number variation.

File Name: Supplementary Data 6

Description: The summary of heterozygosity ratio of 427 samples.

File Name: Supplementary Data 7

Description: 38 long continuous heterozygous SNPs clustered regions of high frequency (high-LCHRs) detected in the moso bamboo population.

File Name: Supplementary Data 8

Description: GO enrichment analysis for the genes which overlap with long continuous heterozygous SNPs clustered regions of high frequency (high-LCHRs).

File Name: Supplementary Data 9

Description: 15 long continuous heterozygous SNPs clustered regions of low frequency (low-LCHRs) detected in the moso bamboo population.

File Name: Supplementary Data 10

Description: GO enrichment analysis for the genes which overlap with long continuous heterozygous SNPs clustered regions of low frequency (low-LCHRs).

File Name: Supplementary Data 11

Description: The potential regions and candidate genes under balancing selection for the moso bamboo population.

File Name: Supplementary Data 12

Description: GO enrichment analysis for the candidate genes under balancing selection.

File Name: Supplementary Data 13

Description: The candidate genes under balancing selection involved in disease resistance or environmental adaptation.

File Name: Supplementary Data 14

Description: The nine traits closely related to bamboo properties were measured.

File Name: Supplementary Data 15

Description: The twelve environmental factors collected on fifteen moso bamboo areas.

File Name: Supplementary Data 16

Description: The potentially associated regions and candidate genes for the trait of bending strength 12°.

File Name: Supplementary Data 17

Description: The potentially associated regions and candidate genes for the trait of compressive strength.

File Name: Supplementary Data 18

Description: The potentially associated regions and candidate genes for the trait of density.

File Name: Supplementary Data 19

Description: The potentially associated regions and candidate genes for the trait of elastic modulus.

File Name: Supplementary Data 20

Description: The potentially associated regions and candidate genes for the trait of ground diameter.

File Name: Supplementary Data 21

Description: The potentially associated regions and candidate genes for the trait of maximum load.

File Name: Supplementary Data 22

Description: The potentially associated regions and candidate genes for the trait of node number.

File Name: Supplementary Data 23

Description: The potentially associated regions and candidate genes for the trait of tensile modulus.

File Name: Supplementary Data 24

Description: The potentially associated regions and candidate genes for the trait of under crown height.

File Name: Supplementary Data 25

Description: Candidate associated genes related to the cell wall, carbohydrate metabolism and environmental adaptation.
