# Supplementary material for: Analysis of 427 genomes reveals moso bamboo population structure and genetic basis of property traits
Source: Nat Commun. 2021 Sep 15;12:5466. doi: 10.1038/s41467-021-25795-x (PMC8443721; doi:10.1038/s41467-021-25795-x)
Supplement: Supplementary file 4 — Reporting Summary [file 41467_2021_25795_MOESM4_ESM.pdf]

nature research

Corresponding author(s): Zhimin Gao

Last updated by author(s): Aug 17, 2021

Reporting Summary

Nature Research wishes to improve the reproducibility of the work that we publish. This form provides structure for consistency and transparency in reporting. For further information on Nature Research policies, see our Editorial Policies and the Editorial Policy Checklist.

Statistics

For all statistical analyses, confirm that the following items are present in the figure legend, table legend, main text, or Methods section.

n/a	Confirmed	
☐	☒	The exact sample size (*n*) for each experimental group/condition, given as a discrete number and unit of measurement
☐	☒	A statement on whether measurements were taken from distinct samples or whether the same sample was measured repeatedly
☐	☒	The statistical test(s) used AND whether they are one- or two-sided<br>*Only common tests should be described solely by name; describe more complex techniques in the Methods section.*
☐	☒	A description of all covariates tested
☐	☒	A description of any assumptions or corrections, such as tests of normality and adjustment for multiple comparisons
☐	☒	A full description of the statistical parameters including central tendency (e.g. means) or other basic estimates (e.g. regression coefficient) AND variation (e.g. standard deviation) or associated estimates of uncertainty (e.g. confidence intervals)
☒	☐	For null hypothesis testing, the test statistic (e.g. *F*, *t*, *r*) with confidence intervals, effect sizes, degrees of freedom and *P* value noted<br>*Give P values as exact values whenever suitable.*
☒	☐	For Bayesian analysis, information on the choice of priors and Markov chain Monte Carlo settings
☐	☒	For hierarchical and complex designs, identification of the appropriate level for tests and full reporting of outcomes
☒	☐	Estimates of effect sizes (e.g. Cohen's *d*, Pearson's *r*), indicating how they were calculated

Our web collection on statistics for biologists contains articles on many of the points above.

Software and code

Policy information about availability of computer code

Data collection	Sequencing data were collected from Illumina/PacBio/Oxford Nanopore sequencing machines. Twelve environmental factors were collected from this paper (doi: 10.13323/j.cnki.j.fafu(nat.sci.).2020.02.008). Map data were collected from ETOPO2v2c Global Gridded 2-minute elevation and bathymetric data in this data library (doi: 10.7289/V5J1012Q). No additional sofware was used to collect data.
Data analysis	Tools and softwares used in this study are SOAPnuke (version 2.1.5); BWA (version 0.7.12-r1039); SAMtools (version 1.3.1);Picard (version 1.105);GATK (version 3.8-1-0-gf15c1c3ef); PLINK (version 1.90);Manta (version 1.6.0); BreakDancer (version 1.1.2);SURVIVOR (version 1.0.7);VCFtools (version 0.1.17); EnrichmentPipeline (https://sourceforge.net/projects/enrichmentpipeline/, version 1.01); Control-FREEC (version v11.5); SweeD (version 4.0.0); BalLeRMix (version 2.2); PHYLP (version 3.6); EIGENSOFT (version 7.2.1); PHYLIP (version 3.6); ggplot2 (version 3.3.0); R (version 3.5.0); ADMIXTURE (version 1.3.0); CLUMPAK (http://clumpak.tau.ac.il, accessed July 2020); Evolview (version 3); ade4 (version 1.7-17); Cytoscape (version 3.70);PSMC (version 0.6.5-r67); SMC++ (version 1.15.4); SNPable (http://lh3lh3.users.sourceforge.net/snpable.shtml, accessed Nov. 2019); BEDTools (version 2.28.0); NUCmer (version4.0.0); stats (version 4.0.4);EMMAX (version beta-07Mar2010); GAPIT (version 3.0); MAGMA (version 1.07bb); mrMLM (version 4.0); GEC (version 0.2); BambooNET (http://bioinformatics.cau.edu.cn/bamboo/, accessed May 1 2021). Customized codes for data analyses are accessible through GitHub https://github.com/BGI-Qingdao/moso_bamboo_resequencing, and are also publicly available in the Zenodo repository https://doi.org/10.5281/zenodo.5201511.

For manuscripts utilizing custom algorithms or software that are central to the research but not yet described in published literature, software must be made available to editors and reviewers. We strongly encourage code deposition in a community repository (e.g. GitHub). See the Nature Research guidelines for submitting code & software for further information.

Data

Policy information about availability of data

All manuscripts must include a data availability statement. This statement should provide the following information, where applicable:

- Accession codes, unique identifiers, or web links for publicly available datasets
- A list of figures that have associated raw data
- A description of any restrictions on data availability

The sequencing data in this study were deposited in the China National GeneBank (CNGB) under accession number CNP0001535 and were also deposited in the NCBI Sequence Read Archive (SRA) under accession number PRJNA755164.
The datasets of twelve environmental factors are provided with this paper (doi: 10.13323/j.cnki.j.fafu(nat.sci.).2020.02.008).
ETOPO2v2c Global Gridded 2-minute elevation and bathymetric data used to draw geographical map are provided in this data library (doi: 10.7289/V5J1012Q).
The source data underlying Figs. 1b-f, 2b, c, 3c, d, 4a-j as well as Supplementary Figs. 2, 3, 4, 5, 6, 7, 8, 9, 10, 11, 12, 13, 14, 15, 17, 18, 19, 20, 21 are provided in the Source Data file.

Field-specific reporting

Please select the one below that is the best fit for your research. If you are not sure, read the appropriate sections before making your selection.

☐ Life sciences ☐ Behavioural & social sciences ☒ Ecological, evolutionary & environmental sciences

For a reference copy of the document with all sections, see nature.com/documents/nr-reporting-summary-flat.pdf

Ecological, evolutionary & environmental sciences study design

All studies must disclose on these points even when the disclosure is negative.

Study description	We sampled and sequenced 427 moso bamboo individuals, and performed the analysis of population genetics, including population structure, distribution patterns of heterozygous genotypes, balancing selection, and demographic history. We adopted the mean values of three independent measurements for the property-related traits, and performed the genome-wide association study to uncover the potential genes related to property-related traits.
Research sample	We sampled 427 moso bamboo individuals covering 15 representative geographic areas, which represent almost all of the moso bamboo habitats in China. 190 samples were remained for the genome-wide association study based on the success of measurements and quality controls.
Sampling strategy	15 representative geographic areas were set to give comprehensive coverage of all geographical regions in which this species is present. Single moso bamboo was collected from nonconsecutive pieces of bamboo forest, or a distance of more than 1 km, taking approximately 23-30 individuals per population to avoid repeated sampling of the same clonal moso bamboo and to reliably estimate population genetic parameters. Preliminary analysis of the moso bamboo collection with micro-satellite markers (Jiang et al., Tree Genet. Genomes 2017) also allowed to determine the samples to include in the current study.
Data collection	The sequenced reads of studied samples were collected by standard methods for DNA isolation, library construction, and sequencing. In brief, the library of the insert size of ~450 bp were constructed from randomly fragmented genomic DNA, and then sequenced on the Illumina sequencing platform with 150 bp paired-end sequencing mode. The nine traits were measured on the department of Biomaterials at ICBR, as supervised by Benhua Fei based on two standard documents: ISO-22157:2019 and GB/T 15780-1995.
Timing and spatial scale	The samples of moso bamboo were collected in August 2015 and August 2016 that covered an extensive scope of all of the moso bamboo habitats in China, and the spatial scale was the ordering of the collection.
Data exclusions	For genome-wide association study, the following samples were excluded (this procedure was pre-established): the samples with a high genetic relatedness based on pairwise identity-by-state (IBS) genetic distance were excluded to avoid introducing bias due to over-represented genotype in these related and duplicate individuals. The variants were excluded (this procedure was pre-established): the SNPs with minor allele frequency < 0.05 and missing-rate more 20% due to low statistic power to detect SNP-phenotype associations, and these SNPs are also more prone to genotyping errors (Marees et al., Int J Methods Psychiatr Res. 2018).
Reproducibility	Population genetics was performed on genomic DNA which is stable in time so that reproducibility was not used in these analyses. The nine property-related traits were measured with three repeats based on two standard documents: ISO-22157:2019 and GB/T 15780-1995.
Randomization	For population genetics, randomization was not relevant to this study. We attempted to collect all representative individuals of 15 representative areas and the accuracy of the study is related to population size and its representation. Association analysis was performed across 190 samples with the covariates including the population structure, kinship, and first three PCs of twelve environmental factors.
Blinding	To avoid unintentional biases, the investigators were blinded in the procedures of sequencing, detecting the sequence variations, and measuring property-related traits with cryptic code numbers. In the procedures of sample collection, analysis of population genetics and association study, the samples were individually labeled and not blinded, but these did not influence the analysis.

Did the study involve field work? ☐ Yes ☒ No

Reporting for specific materials, systems and methods

We require information from authors about some types of materials, experimental systems and methods used in many studies. Here, indicate whether each material, system or method listed is relevant to your study. If you are not sure if a list item applies to your research, read the appropriate section before selecting a response.

Materials & experimental systems

n/a	Involved in the study
☒	☐ Antibodies
☒	☐ Eukaryotic cell lines
☒	☐ Palaeontology and archaeology
☒	☐ Animals and other organisms
☒	☐ Human research participants
☒	☐ Clinical data
☒	☐ Dual use research of concern

Methods

n/a	Involved in the study
☒	☐ ChIP-seq
☒	☐ Flow cytometry
☒	☐ MRI-based neuroimaging
